# Automated quantification of photoreceptor outer segments in developing and degenerating retinas on microscopy images across scales

**DOI:** 10.3389/fnmol.2024.1398447

**Published:** 2024-05-24

**Authors:** Suse Seidemann, Florian Salomon, Karl B. Hoffmann, Thomas Kurth, Ivo F. Sbalzarini, Robert Haase, Marius Ader

**Affiliations:** ^1^Center for Regenerative Therapies Dresden (CRTD), Technische Universität Dresden, Dresden, Germany; ^2^Center for Molecular and Cellular Bioengineering (CMCB), Technische Universität Dresden, Dresden, Germany; ^3^Faculty of Computer Science, Technische Universität Dresden, Dresden, Germany; ^4^Max Planck Institute of Molecular Cell Biology and Genetics, Dresden, Germany; ^5^Center for Systems Biology Dresden, Dresden, Germany; ^6^Core Facility Electron Microscopy and Histology, Technology Platform, Center for Molecular and Cellular Bioengineering (CMCB), Technische Universität Dresden, Dresden, Germany; ^7^DFG Cluster of Excellence “Physics of Life”, Technische Universität Dresden, Dresden, Germany; ^8^Center for Scalable Data Analytics and Artificial Intelligence (ScaDS.AI), Leipzig University, Leipzig, Germany

**Keywords:** photoreceptor outer segment, cone, retinal development, retinal degeneration, supervised machine learning, segmentation, fluorescence microscopy, electron microscopy

## Abstract

The functionality of photoreceptors, rods, and cones is highly dependent on their outer segments (POS), a cellular compartment containing highly organized membranous structures that generate biochemical signals from incident light. While POS formation and degeneration are qualitatively assessed on microscopy images, reliable methodology for quantitative analyses is still limited. Here, we developed methods to quantify POS (QuaPOS) maturation and quality on retinal sections using automated image analyses. POS formation was examined during the development and in adulthood of wild-type mice via light microscopy (LM) and transmission electron microscopy (TEM). To quantify the number, size, shape, and fluorescence intensity of POS, retinal cryosections were immunostained for the cone POS marker S-opsin. Fluorescence images were used to train the robust classifier QuaPOS-LM based on supervised machine learning for automated image segmentation. Characteristic features of segmentation results were extracted to quantify the maturation of cone POS. Subsequently, this quantification method was applied to characterize POS degeneration in “cone photoreceptor function loss 1” mice. TEM images were used to establish the ultrastructural quantification method QuaPOS-TEM for the alignment of POS membranes. Images were analyzed using a custom-written MATLAB code to extract the orientation of membranes from the image gradient and their alignment (coherency). This analysis was used to quantify the POS morphology of wild-type and two inherited retinal degeneration (“retinal degeneration 19” and “rhodopsin knock-out”) mouse lines. Both automated analysis technologies provided robust characterization and quantification of POS based on LM or TEM images. Automated image segmentation by the classifier QuaPOS-LM and analysis of the orientation of membrane stacks by QuaPOS-TEM using fluorescent or TEM images allowed quantitative evaluation of POS formation and quality. The assessments showed an increase in POS number, volume, and membrane coherency during wild-type postnatal development, while a decrease in all three observables was detected in different retinal degeneration mouse models. All the code used for the presented analysis is open source, including example datasets to reproduce the findings. Hence, the QuaPOS quantification methods are useful for in-depth characterization of POS on retinal sections in developmental studies, for disease modeling, or after therapeutic interventions affecting photoreceptors.

## 1 Introduction

Photoreceptor cells are highly specialized, light-sensitive neurons located in the retina. Cones and rods are two different types of photoreceptors that initiate vision in mammalian eyes in dependence on high intensity or dim light, respectively. Human vision particularly relies on cones within the macula facilitating high-acuity day-light and color vision, which is important for tasks such as reading, facial recognition, or driving (Molday and Moritz, 2015; Roska and Sahel, 2018). The mouse retina is specialized for night vision and is therefore more dependent on rod function (Carter-Dawson and Lavail, 1979; Jeon et al., 1998). The functionality of all photoreceptors is highly dependent on their outer segments (POS). These typically cylindrical or conical cell compartments are crucial for light detection in the mammalian eye (Mustafi et al., 2009; Molday and Moritz, 2015). POS represent modified primary cilia that contain a massive amount of light-sensitive pigments that reside in hundreds of stacked membranous discs in rods or aligned plasma membrane evaginations in cones (Mayhew and Astle, 1997; Roepman and Wolfrum, 2007; Gilliam et al., 2012; Hsu et al., 2015; Molday and Moritz, 2015; Goldberg et al., 2016; Rakshit et al., 2017; Chen et al., 2021). In the so-called phototransduction cascade incident, light is efficiently detected and amplified by specialized photopigments and transferred into a biochemical signal by a variety of signaling proteins and ion channels (Roepman and Wolfrum, 2007; Molday and Moritz, 2015; Goldberg et al., 2016). To maintain functionality, POS membranes are constantly phagocytosed by the retinal pigment epithelium (RPE; Hollyfield and Rayborn, 1979; Mazzoni et al., 2014; Almedawar et al., 2020; Kwon and Freeman, 2020; Lakkaraju et al., 2020), while new membranes are generated at the base of the POS (Steinberg et al., 1980; Guerin et al., 1993; Burgoyne et al., 2015; Hsu et al., 2015; Volland et al., 2015; Goldberg et al., 2016). The POS is linked to the inner segment via the connecting cilium (Gilliam et al., 2012; Goldberg et al., 2016; Chen et al., 2021). The light-induced change of the membrane potential (hyperpolarization) is passed on via the cell body to the synapse, affecting neurotransmitter release and thus signaling to secondary neurons, i.e., bipolar and horizontal cells (Tom Dieck and Brandstätter, 2006; Baden et al., 2020).

According to their important function to initiate light perception, damage or loss of POS leads to visual impairment and ultimately blindness. Retinitis pigmentosa, Leber's congenital amaurosis, or age-related macular degeneration belong to a group of currently incurable retinal degenerative diseases characterized by photoreceptor dysfunction and loss (Hartong et al., 2006; Fleckenstein et al., 2021). In order to understand degenerative processes and develop treatments for retinal diseases, a detailed investigation into the structural and morphological changes of POS in development and degeneration is of utmost importance.

POS formation can be analyzed *ex vivo* using extracted retinas as flat mounts and sections (Hollyfield and Rayborn, 1979; Steinberg et al., 1980; Obata and Usukura, 1992; Guerin et al., 1993; Mayhew and Astle, 1997; Burgoyne et al., 2015; Ding et al., 2015; Hsu et al., 2015; Daum et al., 2017; Rakshit et al., 2017; Tu et al., 2019; Corral-Serrano et al., 2020). More recently, the generation of retinal organoids from mouse and human pluripotent stem cells containing high numbers of photoreceptors adds another source to assess early POS formation, though full photoreceptor and thus POS maturation have not been observed yet (Zhong et al., 2014; Gonzalez-Cordero et al., 2017; West et al., 2022; Carido et al., 2023; Völkner et al., 2023). Additionally, POS degeneration was studied in various disease models, particularly using inherited retinal degeneration mouse models (Busskamp et al., 2014; Datta et al., 2015; Veleri et al., 2015; Conley et al., 2018; Baehr et al., 2019; Collin et al., 2020; Corral-Serrano et al., 2020). For the development of gene- or cell-replacement strategies as therapeutic interventions for photoreceptor-related diseases, the (re)formation or neuroprotection of POS is of particular interest as a prerequisite for proper light detection (Assawachananont et al., 2014; Trouillet et al., 2018; Gasparini et al., 2019, 2022; Tu et al., 2019; Aguirre et al., 2021; Ribeiro et al., 2021; Van Gelder et al., 2022; West et al., 2022; Yamasaki et al., 2022). Recent improvements also allow *in vivo* visualization of retinal structures and automated quantification, including POS via high-resolution optical coherence tomography (Rathke et al., 2014; Sonka and Abràmoff, 2016; Wang et al., 2023). However, the current resolution does not allow quantification at the single-cell (or single POS) level or the distinction of photoreceptor subtypes. Hence, the analysis of retinal sections might still be preferable for preclinical analysis in certain cases.

Important insights in outer segment structure and formation have been achieved using qualitative microscopic observations and manual quantification of POS (Carter-Dawson and Lavail, 1979; Hollyfield and Rayborn, 1979; Guerin et al., 1993; Mayhew and Astle, 1997; Rakshit et al., 2017). This includes representative images of immunohistochemical staining of photopigments (e.g., rhodopsin and S- or M/L-opsin), proteins involved in the signal transduction cascade (e.g., recoverin, transducin, arrestin, phosphodiesterase), or structural markers of POS (e.g., peripherin, ROM1; Assawachananont et al., 2014; Daum et al., 2017; Shindou et al., 2017; Tu et al., 2019; Gasparini et al., 2022; West et al., 2022; Yamasaki et al., 2022; Völkner et al., 2023). Electron microscopy or tomography images were used to assess POS structure, volume, disc assembly, and size (Carter-Dawson and Lavail, 1979; Obata and Usukura, 1992; Eberle et al., 2012; Gilliam et al., 2012; Burgoyne et al., 2015; Ding et al., 2015; Goldberg et al., 2016; Daum et al., 2017; Ribeiro et al., 2021; Gasparini et al., 2022). Furthermore, qualitative evidence for outer segment formation after cell transplantation into the retina has been shown in some studies (Eberle et al., 2012; Assawachananont et al., 2014; Ribeiro et al., 2021; Gasparini et al., 2022).

While manual analyses of POS are time-consuming and limit scalability to larger datasets, automated bio-image analysis might be preferable for reproducible and less time-consuming quantitative assessment of POS formation and structure. Additionally, automated systems have the advantage of circumventing possible limitations that are sometimes associated with manual quantifications (e.g., inter- and intra-observer variations). Nowadays, sophisticated models relying on supervised or unsupervised machine learning can be applied to filter images and enhance the contrast or to segment regions of interest from the background (Krull et al., 2019; Haase et al., 2020, 2022; Stringer et al., 2021). Robust models obtained by training and evaluation scale more easily to larger image datasets.

Here, we report two methods to quantify POS formation and quality. First, a supervised machine learning model was established and validated for automated segmentation of POS on images from immunostained cryosections. Subsequently, the number of labels and different size-, shape-, and intensity parameters were extracted from three-dimensional image stacks and quantified. Second, we developed a method to quantify the morphology of POS membrane stacks on transmission electron microscopy (TEM) images. This method uses the image intensity gradient to obtain the orientation of membrane slices and their mutual alignment within stacks and POS, which allows for the automatic quantification of selected ultrastructural details of POS formation and degeneration. We provide evidence that both automated methods can be used to quantitatively assess developmental and degenerative processes in POS. The underlying code and bio-image datasets are published together with further details in an associated GitHub repository (Salomon et al., 2024b) and BioImage Archive repository (Salomon et al., 2024a) to ensure usability in the research fields of retinal development, degeneration, and regeneration, including related therapeutic approaches.

## 2 Methods

### 2.1 Animals

Mouse colonies were bred and maintained at the Center for Regenerative Therapies (CRTD), TU Dresden, mouse facility at a 12 h light/12 h dark cycle with *ad libitum* access to food and water. The wild-type (WT) colony was founded by animals from the C57BL/6JRj strain that were purchased from Janvier.^1^ The “cone photoreceptor function loss 1” (Cpfl1; B6.CXB1-Pde6c^cpfl1^/J, Chang et al., 2002, 2009) mouse colony that shows cone photoreceptor dysfunction/loss was found in mice provided by Bernd Wissinger (Institute of Ophthalmic Research, Tübingen, Germany). “Rhodopsin knock-out” mice (RhoKO; 129.Rho^tm1Ph^; Humphries, 1997) serve as a model for autosomal recessive retinitis pigmentosa. The “retinal degeneration 19” mouse (rd19; B6.BXD83-Prom1^rd19^/BocJ; Jackson laboratories, stock #026803; Chang et al., 2012; provided by Denis Corbeil) provides a model for autosomal recessive retinitis pigmentosa 41 (arRP41) caused by a spontaneous mutation in prominin1, with early-onset photoreceptor degeneration progressing to severe loss in adult animals.

All animal experiments were approved by the ethics committee of the Technische Universität Dresden and the Landesdirektion Sachsen (approval numbers: TVT 5/2018 and TVV 22/2018). All relevant European Union regulations, German laws (Tierschutzgesetz), the Association for Research in Vision and Ophthalmology (ARVO) statement on the Use of Animals in Ophthalmic and Vision Research, and the NIH Guide for the Care and Use of Laboratory Animals were strictly followed for all animal work.

### 2.2 Quantification of photoreceptor outer segment number, size, shape, and intensity parameters on light microscopy images

#### 2.2.1 Collection and preparation of retinal sections

**Figure 1:**
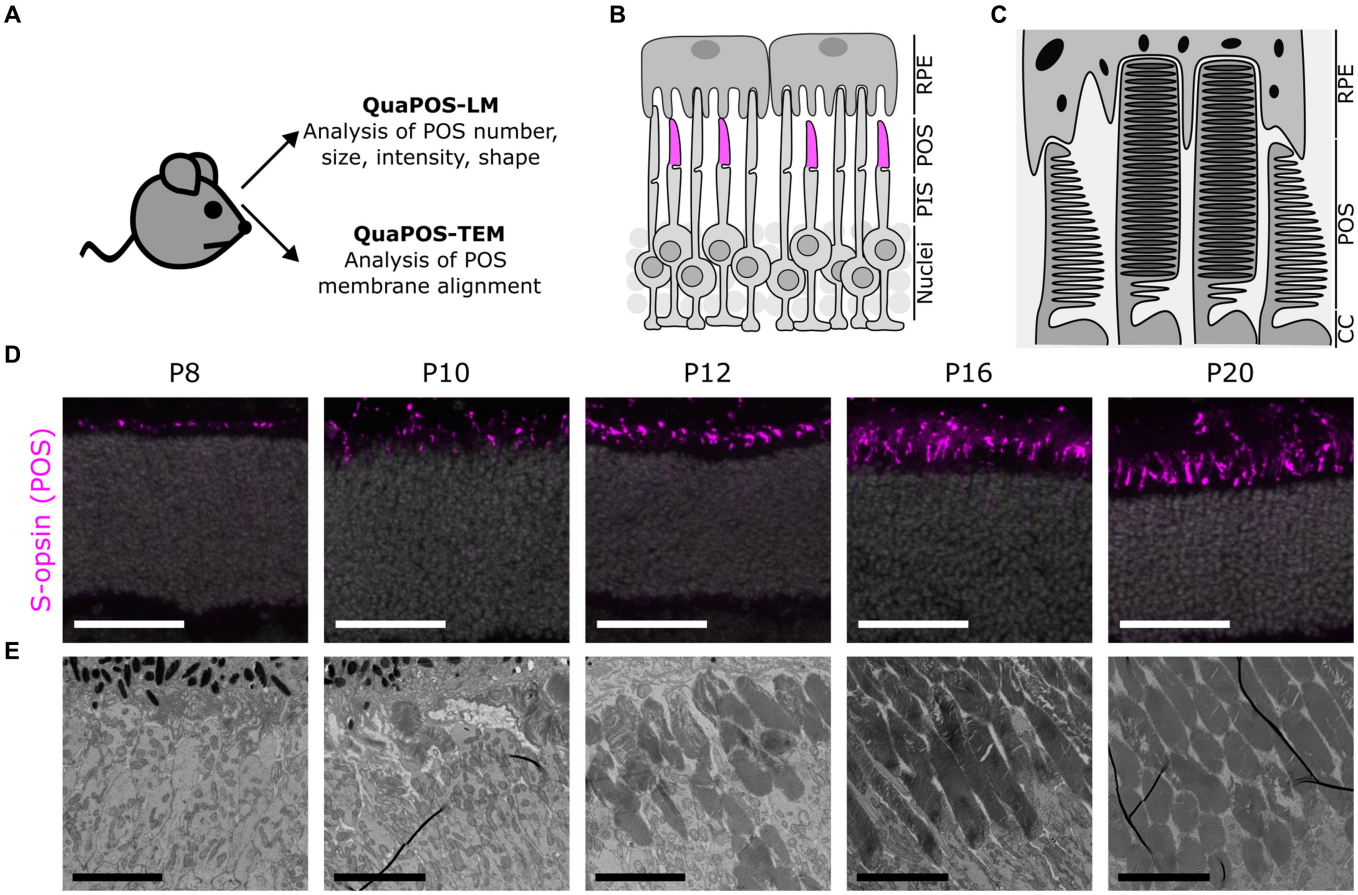
Assessment of photoreceptor outer segments during postnatal mouse development using light (LM) and transmission electron microscopy (TEM) images. **(A)** Experimental outline to develop quantification methods for the analysis of POS on LM and TEM images. Eyes were collected from WT mice (C57BL/6JRj) and processed for analysis of retinal sections via LM or TEM. **(B)** Schematic overview of photoreceptors and retinal pigment epithelial cells (RPE) in the outer retina. Photoreceptor inner segments (PIS) and outer segments (POS) extend apically toward the RPE. Cone POS were immunohistochemically labeled with S-opsin (magenta). **(C)** Ultrastructural scheme of POS as seen in TEM. POS consist of several membrane discs in rods and aligned plasma membrane evaginations in cones. **(D)** Immunohistochemical staining of S-opsin (magenta) and DAPI (gray) on cryosections at different postnatal development stages. Staining indicates a change in size, shape, and number of cone POS throughout postnatal development. Scale bar = 50 μm. **(E)** TEM images of POS at different postnatal development stages. Throughout postnatal development, POS increased in number and size. Later postnatal development stages indicated highly organized POS membrane stacks (scale bar = 5 μm).

Mice were sacrificed at different postnatal ages (postnatal day (P) 8–P245, Figures 1A,D), and eyes were enucleated (as previously described in Gasparini et al., 2022). Subsequently, eyes were fixed in 4% paraformaldehyde (PFA, Sigma-Aldrich, P6148) for light microscopy (LM) analysis at 4°C overnight. Eyes were washed in PBS before the cornea was punctured with a 30 G × 0.5 needle. Cornea, iris, lens, and vitreous were removed with a pair of micro-scissors and curved forceps. The remaining muscle tissue around the sclera was gently removed, and the optic nerve was cut as short as possible.

After dissection, the eyecup containing sclera, choroid, RPE, and retina was cryopreserved overnight at 4°C in a 30% (weight/volume) sucrose (Sigma-Aldrich, S7903) solution and embedded in optimal cutting medium (OCT, NEG50, Thermo Scientific) inside of Tissue-Tek. Samples were sectioned at 20 μm thickness using a CryoStar™ NX70 cryostat. Serial transverse sections were collected on Superfrost™ Plus Adhesion Microscopy Slides, air-dried for 30 min at 37°C, and stored at −70°C.

#### 2.2.2 Immunohistochemistry

Slides with sections for antibody staining were air-dried for 30 min and rehydrated in PBS for 30 min at room temperature (as previously described in Gasparini et al., 2022). During rehydration, slides were covered with Shandon™ cover plates and placed into Shandon™ Sequenza™ racks. Next, slides were incubated in blocking buffer (0.3% Triton X-100, 5% Donkey or Goat Serum, and 1% BSA [Serva GmBH, 11926.04] dissolved in PBS) for 1 h at room temperature. Afterwards, the sections were incubated with primary antibodies (Rabbit-Anti-Peripherin2, Thermo Scientific, 1:200; Goat-Anti-S-opsin, Santa Cruz, sc-14363, 1:200) dissolved in blocking buffer at 4°C for 12 h. The slides were kept at room temperature for 30 min and washed with PBS. Afterwards, the tissue was incubated with secondary antibodies (Donkey-Anti-Rabbit-Cy2, Jackson IR, 711-225-152; Donkey-Anti-Goat-Cy3, Jackson IR, 705-165-147) and DAPI nuclear staining solution (1:5,000) dissolved in PBS. Then, slides were washed extensively in PBS and water before mounting with Aqua-Poly/Mount (Polysciences, 18,606) and a 24 × 50 mm #1.5 coverslip. Finally, slides were stored at 4°C after air drying at room temperature in a dark place.

#### 2.2.3 Light microscopy image acquisition

Images of the central retina (maximum 400 μm distance to the optic nerve) were acquired with an upright Zeiss Apotome Imager Z2 (Apotome) and ZEN 3.5 blue edition software. To avoid overexposure of POS, the exposure times were normalized accordingly to their lowest value in one round of staining and kept constant within the experiment. Three-dimensional images were acquired with the Z-stack function of the program using a 20× air objective. The different channels were split into individual images using the Fiji/ImageJ Macro. Subsequently, image and statistical analyses were carried out with python 3.9 and devbio-napari 0.8.1.

#### 2.2.4 Software training of the pixel classifier QuaPOS-LM for cone outer segments on light microscopy images

##### 2.2.4.1 Image pre-processing

Normalization was carried out to standardize the background and intensity across all images. The background was subtracted by a top-hat filter with the radius set to 3 in all three dimensions and an according function from the pyclesperanto-prototype package 0.23.2 (Haase et al., 2023). Subsequently, the intensity of all images was normalized based on the maximum intensity of each image with the following formula:


$$normalized\;image=\frac{image}{maximum\;intensity\;of\;image}\times 4096$$

##### 2.2.4.2 Training of a pixel classifier

To train a pixel classifier that segments cone POS, images from retinal sections stained for S-opsin at seven timepoints (P8, P10, P12, P14, P16, P20, and P24) were divided into a training and test dataset. The training dataset contained 21 images (three biological replicates per timepoint). The test dataset contained seven images (one biological replicate per timepoint). The training dataset was used to train different machine learning models. The test dataset was used to calculate the performance of the models.

**Figure 2:**
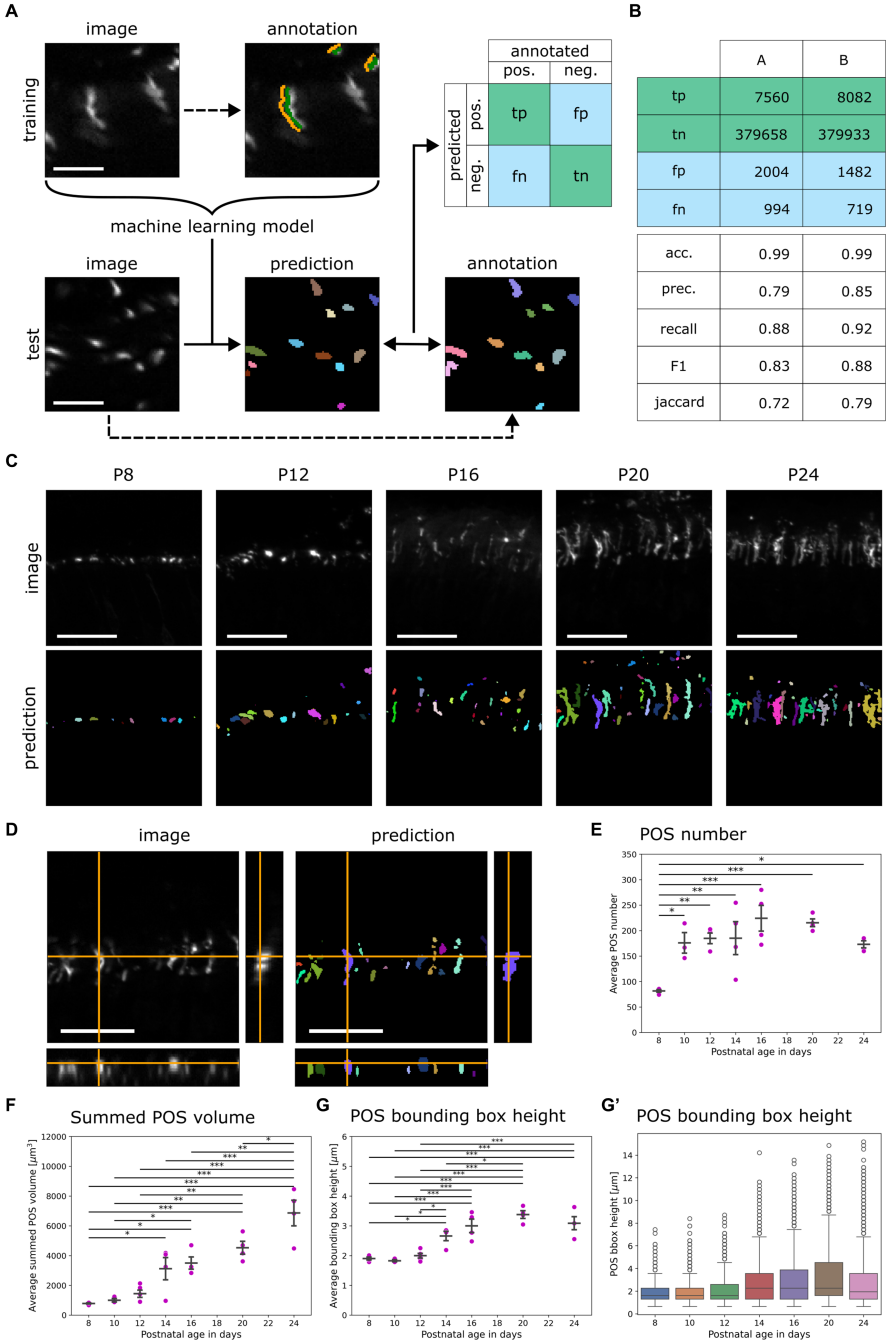
Development of the random forest classifier QuaPOS-LM for quantification of S-cone POS. **(A)** A random forest pixel classifier to automatically distinguish POS from background was trained and validated. The background (orange) and the POS signal (green) of cones were sparsely annotated on LM images stained for S-opsin in a training dataset by a human. The dataset was used to train a machine learning (ML) model that automatically separated the signal of cone POS from its background. On a test dataset, manual annotations of two persons A and B were compared to the prediction of the ML model to compute a confusion matrix containing true positive (tp), true negative (tn), false positive (fp), and false negative (fn) pixel counts (scale bar = 10 μm). **(B)** The confusion matrix was computed with test annotations from two different people. The performance of the random forest classifier was estimated by calculating different scores. The accuracy (acc.), precision (prec.), recall, F1-score (F1), and Jaccard Index (Jaccard) were calculated with the obtained values from the confusion matrix. **(C)** QuaPOS-LM can be applied to predict the labels from S-opsin-stained croysections at different postnatal development stages (P8–P24; scale bar = 25 μm). **(D)** Inspecting the orthogonal view of an intensity image and its corresponding prediction showed that QuaPOS-LM can predict S-opsin-stained cone POS in 3D image stacks (scale bar = 25 μm). **(E)** POS number increased throughout postnatal development. The number of POS was estimated by the number of predicted labels in each image. The average number of POS per timepoint was computed from the average values of respective biological replicates (mean ± SEM, *n* = 3–5, *N* = 3, one-way ANOVA followed by *post-hoc* Tukey test, * *p* < 0.05, ** *p* < 0.01, *** *p* < 0.001). **(F)** The summed POS volume increased throughout postnatal development. The summed POS volume was determined in each image and averaged by the biological replicate. The average summed POS volume was computed from the biological replicates (mean ± SEM, *n* = 3–5, *N* = 3, one-way ANOVA followed by *post-hoc* Tukey test, * *p* < 0.05, ** *p* < 0.01, *** *p* < 0.001). **(G,G′)** The bounding box height increased throughout postnatal development. **(G)** The average POS bounding box height was determined in each image. Afterwards, the average per biological replicate was determined (mean ± SEM, *n* = 3–5, *N* = 3, one-way ANOVA followed by *post-hoc* Tukey test, * *p* < 0.05, ** *p* < 0.01, *** *p* < 0.001). **(G′)** A boxplot representation of the POS bounding box height. Here, all measured POS were pooled according to age, independent of the biological replicate. Whiskers represent the 1.5 interquartile range. An increase in the bounding box height was observed.

The Z-stack images in the training dataset were normalized, and the region containing S-opsin signal was cropped and randomly renamed to secure blinded analysis. Afterwards, 100 pixels in each class (signal or background) per image of the training dataset were annotated as ground truth in the software napari according to intensity as well as the structure of the POS (Figure 2A).

Subsequently, the images were used to train different random forest binary pixel classifiers that distinguish signal from background using the accelerated pixel and object classification (APOC) 0.12.0 package (Haase et al., 2022). The number of decisions per decision tree was set to 2, and the number of decision trees in the classifier was 100. Different filters were applied to the images in the training dataset while the classifiers were trained: original image, Gaussian blur, difference of Gaussian blur, Laplace box of Gaussian blur, Sobel of Gaussian blur, median box, mean box, and top-hat box, all with a respective sigma of 1 or 2. As a result, a code was produced that is able to predict a set of labels corresponding to S-opsin positive regions (cone POS) for provided images. We named this code QuaPOS-LM. The code and the bio-image datasets are provided in the respective GitHub repository (Salomon et al., 2024a, https://github.com/FloskelSalomon/quapos/tree/main) and BioImage Archive repository (Salomon et al., 2024a).

##### 2.2.4.3 Validating the performance of the QuaPOS-LM classifier

To validate the performance of the trained classifier QuaPOS-LM, S-opsin positive regions (cone OS) of one image per timepoint (test dataset) were fully annotated in three Z-sections by two persons (Figure 2A). Afterwards, the same images were analyzed by QuaPOS-LM, and a set of segmentation results was predicted (Figure 2A). Manual annotations were then compared with the QuaPOS-LM predictions, and a confusion matrix was computed with a function from the segmentation game package 0.2.0 (Figure 2A, Haase, 2022). The confusion matrix contained the number of true positive (TP), true negative (TN), false positive (FP), and false negative (FN) pixel counts (Figures 2A,B). Subsequently, precision, accuracy, recall, Jaccard-index, and F1-score were determined with the following equations:


$$accuracy=\frac{TP+TN}{TP+TN+FP+FN}$$

$$precision=\frac{TP}{TP+FP}$$

$$recall=\frac{TP}{TP+FN}$$

$$F1\_score=2\times \frac{precision\times recall}{precision+recall}$$

$$Jaccard\_index=\frac{TP}{TP+FP+FN}$$

#### 2.2.5 Feature extraction of POS segmentations predicted by QuaPOS-LM

To quantify cone POS retinal images, three biological replicates with 1–3 technical replicates were normalized and provided to QuaPOS-LM to predict POS labels. Afterwards, images were rescaled so that pixels in all dimensions corresponded to the same size (1 pixel = 0.323 μm). Following segmentation, features of the predicted labels were extracted as quantitative measurements using the packages napari simpleitk 0.4.5 (Haase, 2023) and porespy 2.3.0 (Gostick et al., 2019). The number of predicted POS and their corresponding size, shape, and intensity were measured.

#### 2.2.6 Statistical analyses of POS labels predicted by QuaPOS-LM

Labels in the 5–95% confidence interval (according to label volume) were considered specific signals and included in the statistical analysis. Statistical analysis was carried out with scipy 1.10.1 (Virtanen et al., 2020) and statsmodels 0.13.5 (Seabold and Perktold, 2010). Features were averaged in their respective biological replicates and conditions. Pearson’s R correlation coefficient of extracted features was computed as correlation matrix with pandas (The Pandas Development Team, 2024). Subsequently, a one-way analysis of variance (one-way ANOVA) and post-hoc Tukey test were conducted for selected features. Statistical comparison between WT and Cpfl1 mutant mice was carried out with a Welsh’s *t*-test. The null hypothesis was rejected when the *p*-value was below 0.05. Plots were computed with seaborn 0.12.2 (Waskom, 2021) and matplotlib 3.7.0 (Caswell et al., 2024).

### 2.3 Quantification of photoreceptor outer segment membrane stack alignment and morphology on transmission electron microscopy images

#### 2.3.1 Collection, sample preparation, resin embedding, ultramicrotomy, and transmission electron microscopy

Mice were sacrificed at different postnatal ages (P8–P30, Figures 1A,E), and eyes were enucleated and dissected as described in 2.2.1. For TEM analyses, eyes were prefixed in 4% formaldehyde (prepared from paraformaldehyde prills, Electron Microscopy Sciences [EMS] # 19208) in 100 mM phosphate buffer (PB), washed in PBS, further dissected to small (0.5–1 mm) blocks, and postfixed with modified Karnovsky’s fixative (2% PFA/2% glutaraldehyde (EMS #16220) in 50 mM HEPES (pH 7.4, EMS #16782) or in 100 mM PB) at 4°C overnight (Kurth et al., 2010; Gasparini et al., 2022; Völkner et al., 2023). After aldehyde fixation, the tissue pieces were washed and further postfixed in 2% aqueous OsO_4_ solution containing 1.5% potassium ferrocyanide and 2 mM CaCl_2_, followed by several washes in water, incubation in 1% thiocarbohydrazide, washes in water, and a second contrasting step in 2% osmium/water (OTO procedure, Hanker et al., 1966; Deerinck et al., 2010). After several washes in water, the samples were en-bloc contrasted with 1% uranyl acetate/water, washed again in water, dehydrated in a graded ethanol series up to 100% ethanol over molecular sieve, and infiltrated with the Epon substitute EMBed 812. After embedding, samples were cured at 65°C overnight. 70 nm thin sections were cut with a Leica UC6 ultramicrotome and collected on formvar-coated slot grids. Sections were stained with lead citrate (Venable and Coggeshall, 1965) and uranyl acetate and imaged on a Jeol JEM1400 Plus (camera: Ruby, JEOL), both running at 80 kV acceleration voltage. Montages (3 × 3 images) were prepared using the automated montaging function of the ruby camera software.

#### 2.3.2 Selection of ROIs for QuaPOS-TEM analysis

TEM images acquired at 5000× magnification (287.5 pixels = 1 μm) were used to analyze the POS ultrastructure through membrane stack alignment and morphology. Single POS were selected as separate regions of interest (ROIs) using the Selection Brush Tool in ImageJ (version 1.54b). Cone and rod POS were not distinguished. POS were identified as subcellular structures with electron-dense membranes at the tip of a connecting cilium or between the mitochondria-rich inner segments and the highly pigmented RPE. POS were selected randomly per experimental condition (19–79 POS from at least six different images of 1–3 biological replicates). Regions showing obvious disruptions caused by tissue processing or image montage were not considered. All ROIs of one image were analyzed individually but saved together for analysis per specimen.

#### 2.3.3 Description of custom code for QuaPOS-TEM analysis

**Figure 3:**
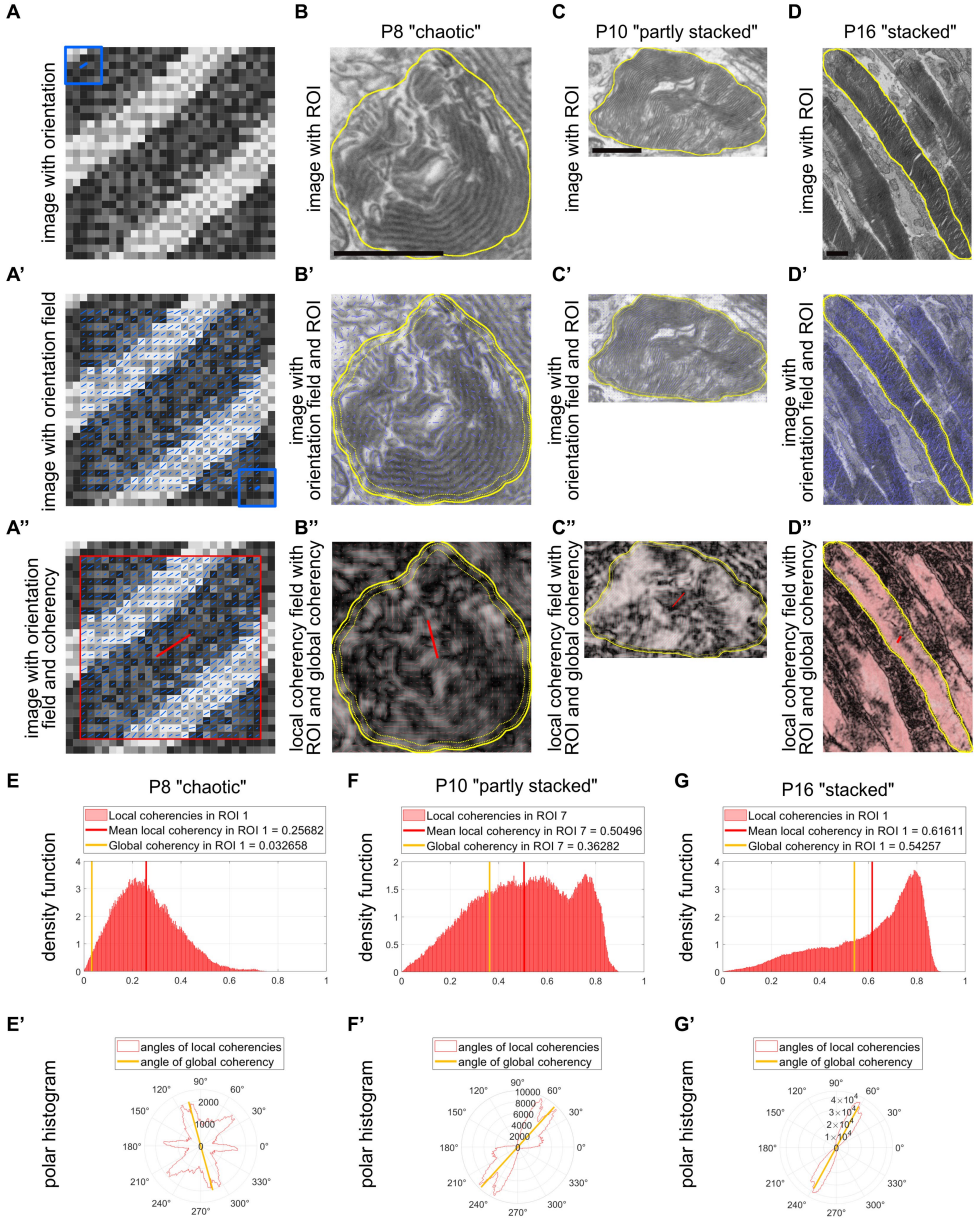
QuaPOS-TEM can efficiently quantify the coherency of POS disc membranes. **(A,A′)** Using electron microscopic micrographs, QuaPOS-TEM calculates the orientations (blue sticks) as nematic directors based on the image intensity gradient within a sliding window of 5 × 5 pixels (blue box) centered around the query point. This orientation field tracks the underlying membranous structures **(A′)**. **(A″)** Subsequently, the coherency (degree of alignment) of the orientations is computed in a box of 25 × 25 pixels (red box, local alignment) or across complete ROIs (global alignment). The coherency is represented as a red stick with a certain length and direction. **(B–D)** POS of WT mice of different developmental ages (P8, P10, and P16) showed various membrane morphologies on TEM images. Scale bar = 1 μm. **(B′–D′,B″–D″)** Within selected ROIs (yellow contour), the orientation of the membranes was tracked and their coherency was computed. Local coherencies were visualized as thin red sticks of a certain length and angle and emphasized by background color **(B″–D″)**. The higher the local coherency was, the longer the stick and the brighter the background. Global coherencies were shown as thick red sticks **(B″–D″)**. **(E–G)** Density functions revealed the distribution of local coherencies (red areas) and the resulting mean local coherency (thick red line) and global coherency (thick orange line). The “chaotic” POS **(E)** showed low, the “partly stacked” POS **(F)** medium, and the “stacked” POS **(G)** high values. **(E′,F′,G′)** The directions of local coherencies within each ROI were plotted as polar histograms (red outlines) respecting the 180-degree symmetry and showed the computed angle of the global coherency (thick orange line). The polar histogram of the “chaotic” POS **(E′)** showed a variety of different coherency directions, while the “partly stacked” POS **(F′)** and “stacked” POS **(G′)** corresponded to narrow distributions of coherency angles.

A custom MATLAB code was developed to analyze the orientations of membrane stacks in TEM images and their coherency, that is, the degree of alignment between membranes both locally and globally across individual POS. The code, respective explanations for usage, and the bio-image datasets can be found in the respective GitHub repository (Salomon et al., 2024b) and BioImage Archive repository (Salomon et al., 2024a). Briefly, all ROIs (POS) within each image are processed concurrently by the following steps:

1 Orientation analysis based on the image intensity gradient, using the convolutional Scharr operator optimized for minimal orientation bias (Scharr, 2000, Tabelle B.11, operator “5 × 5-opt” with angular error below 0.00007 degrees) within a sliding window of 5 × 5 pixels centered around the query point (Figures 3A,A′, blue sticks in blue boxes). The local orientation of individual membrane layers is given by the orientation of small-scale high-intensity image structures and is therefore perpendicular to the image intensity gradient. Note that orientations are nematic directors, that is, they are invariant under 180-degree rotation (whereas usual polar vectors are invariant only after 360-degree rotation). Therefore, plots show orientations as short blue sticks (without the arrowhead that polar vectors would have), and internal representation of nematic orientations uses either of two antipodal polarities (Figures 3A,B′–D′). A gradient vector $\left(v_{x},v_{y}\right)$ defines the orientation represented by $\left(v_{y},-v_{x}\right),$ or by $\left(-v_{y},v_{x}\right)$ . Alternatively, orientations are represented by a magnitude $m=\sqrt{v_{x}^{2}+v_{y}^{2}}$ and an orientation angle θ determined up to additive multiples of 180-degree (or π radians) by $v_{x}=m\;\sin\theta ,v_{y}=m\;\cos\theta$ . This way, the gradient’s magnitude becomes a weighting factor m . All subsequent analysis is independent of the specific choice of representation.
2 Coherency analysis measures the degree of alignment between nematic vectors. The coherency for orientations within some neighborhood N from the Q -tensor

$$\begin{array}{l} Q=\underset{x\;\mathrm{in}\;N}{\sum }m\left(x\right)\left(\begin{array}{cc} \cos^{2}\theta \left(x\right)-0.5 & \cos\theta \left(x\right)\sin\theta \left(x\right)\\ \cos\theta \left(x\right)\sin\theta \left(x\right) & \sin^{2}\theta \left(x\right)-0.5 \end{array}\right)\\ \;\;\;\;\;=\left(\begin{array}{cc} Q_{xx} & Q_{xy}\\ Q_{xy} & -Q_{xx} \end{array}\right) \end{array}$$

is computed as twice its larger eigenvalue $2\;\sqrt{Q_{xx}^{2}+Q_{xy}^{2}}$ which is also known as the *scalar nematic order parameter* (Soans et al., 2022).

3 Choosing the neighborhood N as a box of 25 × 25 pixels (box radius = 12) centered around a query point yields the local coherency at that point (Figure 3A″, red stick in red box and Figures 3B″–D″, thin red sticks with length corresponding to coherency value). It measures the alignment of membrane stacks in the vicinity. Averaging over all those query points where the neighborhood and underlying gradient estimation kernels were fully contained within a given ROI defines the mean local coherency of that ROI (Figures 3B″–D″, mean of thin red sticks inside of inner dashed yellow contour).
4 Choosing the neighborhood N as all pixels for which the 5 × 5 kernel of gradient estimation was fully contained within a given ROI (Figures 3B′–D′, outer dashed yellow contour) defines the global coherency of that ROI. For comparison to local coherencies, it is shown in Figures 3B″–D″ as thick red stick. The global coherency measures the alignment of membrane stacks across the complete ROI. The angle of the global coherency was retrieved as the fourth quadrant inverse tangent of the global coherency vector (Figures 3E′–G′).
5 Local coherencies for each query point within an ROI and global coherency as a single nematic vector per ROI are visualized as sticks representing orientations, with coherency as magnitude and direction given by the larger eigenvector of the Q -tensor to its larger eigenvector (Figures 3A″–D″, thin and thick red sticks). Local coherencies within each ROI are also plotted as polar histograms respecting the 180-degree symmetry and as density functions (Figures 3E–G,E′–G′).

For processing different image resolutions, images can be optionally down-sampled, either at the level of the input grayscale image or at the level of orientations, using the Q -tensor to calculate the orientation for each super-pixel from the input pixel orientations.

Following coherency analysis per ROI, the overall alignment per biological replicate was computed. This alignment and the dominant direction of the membranes were calculated from the angles of global coherency (of all ROIs in one biological replicate of one round of imaging) by the Q -tensor, respecting their periodicity of 180 degrees (or π radians).

#### 2.3.4 Statistical analyses of outer segment membrane stack alignment and morphology measured by QuaPOS-TEM

Statistical analyses were performed with GraphPad Prism 9.5.1. One-way analysis of variance (one-way ANOVA) between groups and a subsequent *post-hoc* Tukey multiple comparison test was conducted. The null hypothesis was rejected when the *p*-value was below 0.05. Plots were generated with GraphPad Prism 9.5.1.

## 3 Results

### 3.1 Development of photoreceptor outer segments was assessed on light and transmission electron microscopy images

OS formation was examined during the development of WT mice using retinas analyzed at different postnatal ages (postnatal day P4–P24) on transverse sections via LM and TEM (Figures 1A–E; Supplementary Figure S1).

A comparison of different markers for POS structure and proteins involved in the phototransduction cascade revealed that the marker S-opsin was best suited for establishing the quantification method QuaPOS-LM. The respective staining had a high signal-to-noise ratio, and the structure of separate POS was clearly visible (Figures 1B,D; Supplementary Figure S1A). Major changes during the development of the POS appeared between P6 and P16 (Figure 1D; Supplementary Figure S1A) when POS (qualitatively) increased in number and size.

For ultrastructural characterization of POS formation and structure, TEM images were obtained (Figures 1C,E; Supplementary Figure S1B). At P4 and P6, no structures resembling POS could be observed (Supplementary Figure S1B). Scattered and small POS that show unorganized membrane stacks appeared at P8. At P10 and P12, an increased number and elongation of POS and improved alignment of their membrane stacks were observed. Further thickening of the POS layer took place until P16 and P20 (Figure 1E; Supplementary Figure S1B). In accordance with the immunohistochemical analyses, major changes in the number and structure of the POS appeared between P6 and P16. The obtained images were used to establish the ultrastructural quantification method QuaPOS-TEM through POS membrane alignment, morphology, and orientation.

### 3.2 The automated pixel classifier QuaPOS-LM was developed to detect and quantify cone outer segments

#### 3.2.1 Development of the pixel classifier QuaPOS-LM

To quantify S-opsin positive cone POS on LM images in an automated way, a supervised machine learning approach was used. Before training the model, three-dimensional image stacks of a developmental time series (P8–P24) of C57BL/6JRj mouse retinas were normalized and annotated manually into positive (S-opsin-positive) and negative (background) classes (Figure 2A). These annotated images were used to train different random forest pixel classifiers that distinguish signal from background using the accelerated pixel and object classification (APOC) 0.12.0 package (Haase et al., 2022). During the training of the machine learning model, different combinations of image filter operations were applied to images in the training dataset. Applying the filter combination of Gaussian blur, difference of Gaussian blur, and Laplace box of Gaussian blur (all with a respective sigma of 1) was most efficient as it used a small number of filters, corresponding to low computational effort (Supplementary Figures S2A,B).

The different classifiers were applied to a set of test images, and the corresponding prediction was compared to human annotation (Figure 2A). The computation of different scores revealed a performance of at least 70% within human agreement (Figure 2B). Small and large POS were detected. All classifiers predicted similar results (data not shown).

The chosen classifier predicted ROIs for S-opsin-positive staining on provided images throughout postnatal development as well as in three dimensions (Figures 2C,D). The classifier QuaPOS-LM is available in the corresponding GitHub repository (Salomon et al., 2024b).

#### 3.2.2 Analysis of cone POS predicted by QuaPOS-LM revealed an increase in number and size throughout postnatal development

The classifier QuaPOS-LM was applied to images to quantify changes in POS throughout postnatal development (P8–P24) in WT mice. Corresponding to the LM images, the classifier predicted small, circular POS in early development, which increased in size and became more elongated over time (Figure 2C). However, in later developmental stages, the segmentation predicted structures that corresponded to several POS (here referred to as “clusters”) as well as small objects that may have arisen from high pixel intensities in the background (Supplementary Figure S2C). To exclude clusters and small objects in the analysis, a threshold based on the 5th and 95th percentiles for the volume was computed and applied to the dataset, except when the summed POS volume was analyzed.

The qualitative LM analysis suggested a change in POS number, size, and shape. To investigate these changes, the number of POS as well as size, shape, and intensity parameters were extracted using napari-simpleitk-image-processing 0.4.5 (Haase, 2023) and porespy 2.3.0 (Gostick et al., 2019). The number of POS per analyzed image (located at 200–400 μm of ONL around the optic nerve) depended significantly on the developmental age. A 2-fold increase in POS number was observed between P8 and P24 (Figure 2E). Additionally, POS showed a significant change in size during postnatal development. An 8-fold increase in the summed POS volume as well as an increase of 1 μm in the POS bounding box height (maximum elongation in the *y*-direction) was observed between P8 and P24 (Figures 2F,G). Additionally, analyzing the distribution of the bounding box height of POS over time with a boxplot showed a maximum of around 14 μm as well as an increase in the 75th percentile along with an increase in the 1.5 interquartile range (Figure 2G′).

To visualize the relationships between all measured features, a correlation matrix containing all Pearson’s R correlation coefficients was computed from measurements averaged within their biological replicate (Supplementary Figure S3A). When assessing dependencies over time, it was observed that different features correlated positively or negatively over time, reaching correlation coefficients from −0.7 to 0.9 (Supplementary Figure S3A′). In particular, different size features correlated positively with the postnatal development age. Based on the correlation analysis, the Feret diameter (Supplementary Figures S3B,B′), the minimum intensity (Supplementary Figures S3C,C′), and the sphericity (Supplementary Figures S3D,D′) were further investigated exemplarily and showed changes throughout postnatal development.

Finally, different measurements were exemplarily annotated with their corresponding QuaPOS-LM prediction as a heatmap. Analyzing the POS volume and bounding box height revealed that long POS with a large volume were regularly detected in adults. However, smaller POS with a little volume were frequently observed in adults as well (Supplementary Figures S3E,F). Furthermore, the minimum intensity of properly formed POS did not show any notable difference from immature POS at earlier developmental stages (Supplementary Figure S3G). Finally, the sphericity of POS decreased when comparing early and late development stages (Supplementary Figure S3H).

#### 3.2.3 Quapos-LM revealed a decrease in cone POS number and volume in the Cpfl1 mouse model over time

**Figure 4:**
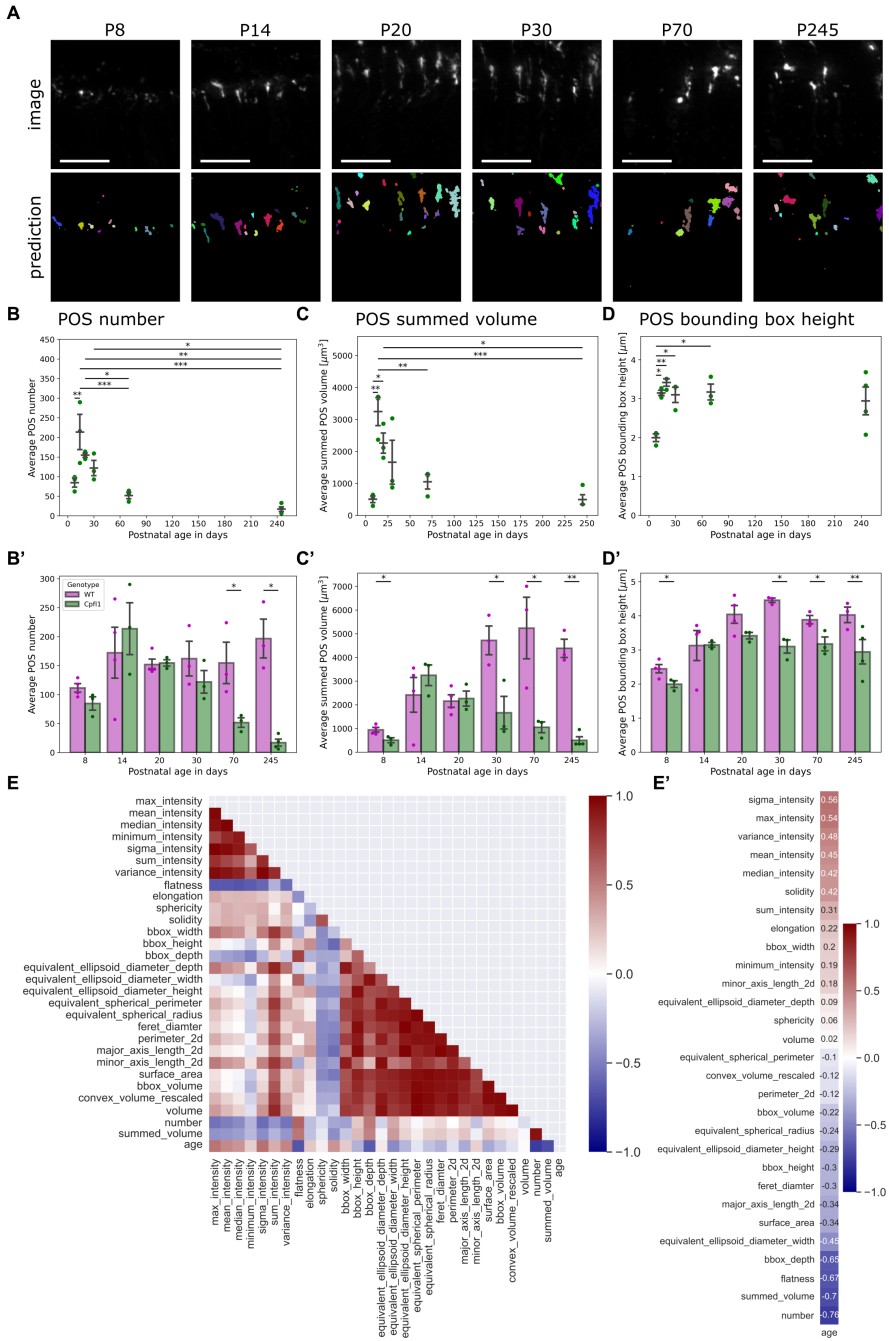
QuaPOS-LM revealed a decrease in cone POS number and volume in a cone degeneration mouse model over time. **(A)** QuaPOS-LM predicted POS on a dataset of S-opsin-stained retinal images from cone photoreceptor function loss 1 mice (Cpfl1) over time (P8–P245) (Scale bar = 25 µm). **(B,B′)** Cpfl1 mice showed a decline in POS number with increasing age. **(B)** Analyzing the Cpfl1 dataset alone revealed an increase in POS number from P08–P14 and a decline until P245 (mean ± SEM, *n* = 3–4, *N* = 1, one-way ANOVA followed by a *post-hoc* Tukey test, * *p* < 0.05, ** *p* < 0.01, *** *p* < 0.001. **(B′)** In comparison to age-matched WT control animals, Cpfl1 mice showed a reduction of POS at P70 and P245 (mean ± SEM, *n* = 3–4, *N* = 1, independent *t*-test, * *p* < 0.05). **(C,C′)** Cpfl1 mice showed a reduction of the summed POS volume with increasing age. The summed POS volume was determined as the summed volume of each image. **(C)** Analyzing the Cpfl1 dataset alone revealed an increase in the summed POS volume from P8 to P14, followed by a decline until P245 (mean ± SEM, *n* = 3–4, *N* = 1, one-way ANOVA followed by a *post-hoc* Tukey test, * *p* < 0.05, ** *p* < 0.01, *** *p* < 0.001). **(C′)** Cpfl1 mice showed significant differences in the summed POS volume at P30, P70, and P245 in comparison to age-matched WT control animals (mean ± SEM, *n* = 3–4, *N* = 1, independent *t*-test, * *p* < 0.05, ** *p* < 0.01). **(D)** Cpfl1 animals showed an increase in the POS bounding box height from P8 to P24. Afterwards, the POS bounding box height remained at 3 μm (mean ± SEM, *n* = 3–4, *N* = 1, one-way ANOVA followed by a *post-hoc* Tukey test, * *p* < 0.05, ** *p* < 0.01, *** *p* < 0.001). **(D′)** In comparison to age-matched WT controls, Cpfl1 animals showed a significant decrease in the POS bounding box height at the ages P8, P30, P70, and P245 (mean ± SEM, *n* = 3–4, *N* = 1, independent *t*-test, * *p* < 0.05, ** *p* < 0.01). **(E)** A heatmap representation of the correlation matrix showed the relationships between all measured features. Pearson’s R correlation coefficients were calculated from averaged values, excluding P8. **(E′)** Correlation vector of the age alone, computed from the correlation matrix. The correlation vector showed the Pearson’s R correlation coefficient of all measured features with age.

Confirming the application of QuaPOS-LM to characterize POS throughout postnatal development, it was of interest to investigate whether the same model could be applied to quantify cone photoreceptor degeneration. The mouse model Cpfl1 is characterized by dysfunction and loss of cone photoreceptors in adults but has not been systematically quantified (Chang et al., 2002, 2009). Qualitative analysis showed a decline of POS in Cpfl1 mice at P70 and P245 as well as aberrant morphology in comparison to age-matched healthy WT control animals (Supplementary Figures S4A,B). Applying QuaPOS-LM to images of Cpfl1 mouse retinal sections stained with S-opsin revealed that the classifier was able to predict POS throughout degeneration in the present phenotype (Figure 4A). Analogous to the analysis of POS during postnatal development, the number of POS as well as their size, shape, and intensity parameters were extracted from the Cpfl1 dataset. Analyzing the number as well as the summed volume per image showed two phases throughout time. First, the number and summed volume of POS increased from P8 to P14. Afterwards, both measurements showed a decrease until P245 (Figures 4B,C). Moreover, in comparison to age-matched WT control animals, a significant decline in POS number in adult Cpfl1 animals was observed (Figure 4B′). Additionally, a significant difference in the summed POS volume was observed at P8 as well as at older stages P30, P70, and P245 (Figure 4C′).

However, the POS bounding box height showed a different trend; it increased similarly between P8 and P14 but stagnated afterwards until P245 (Figure 4C). However, in comparison to age-matched WT control animals, the bounding box height of Cpfl1 mice showed a significant difference at P8, P30, P70, and P245 (Figure 4C′).

Finally, the Pearson’s R correlation coefficients between all features were computed for the Cpfl1 dataset from P14 onwards to investigate the state of degeneration alone (Figure 4E). Analyzing the relationships with age showed that POS number as well as their summed volume decreased over time (Figure 4E′). However, whereas intensity parameters showed a positive correlation with age, indicating that POS in the degeneration model expressed more S-opsin, some measurements regarding size showed a slightly negative correlation.

### 3.3 The automated method QuaPOS-TEM was developed to quantify the coherency of photoreceptor outer segment membranes

#### 3.3.1 Development of QuaPOS-TEM

**Figure 5:**
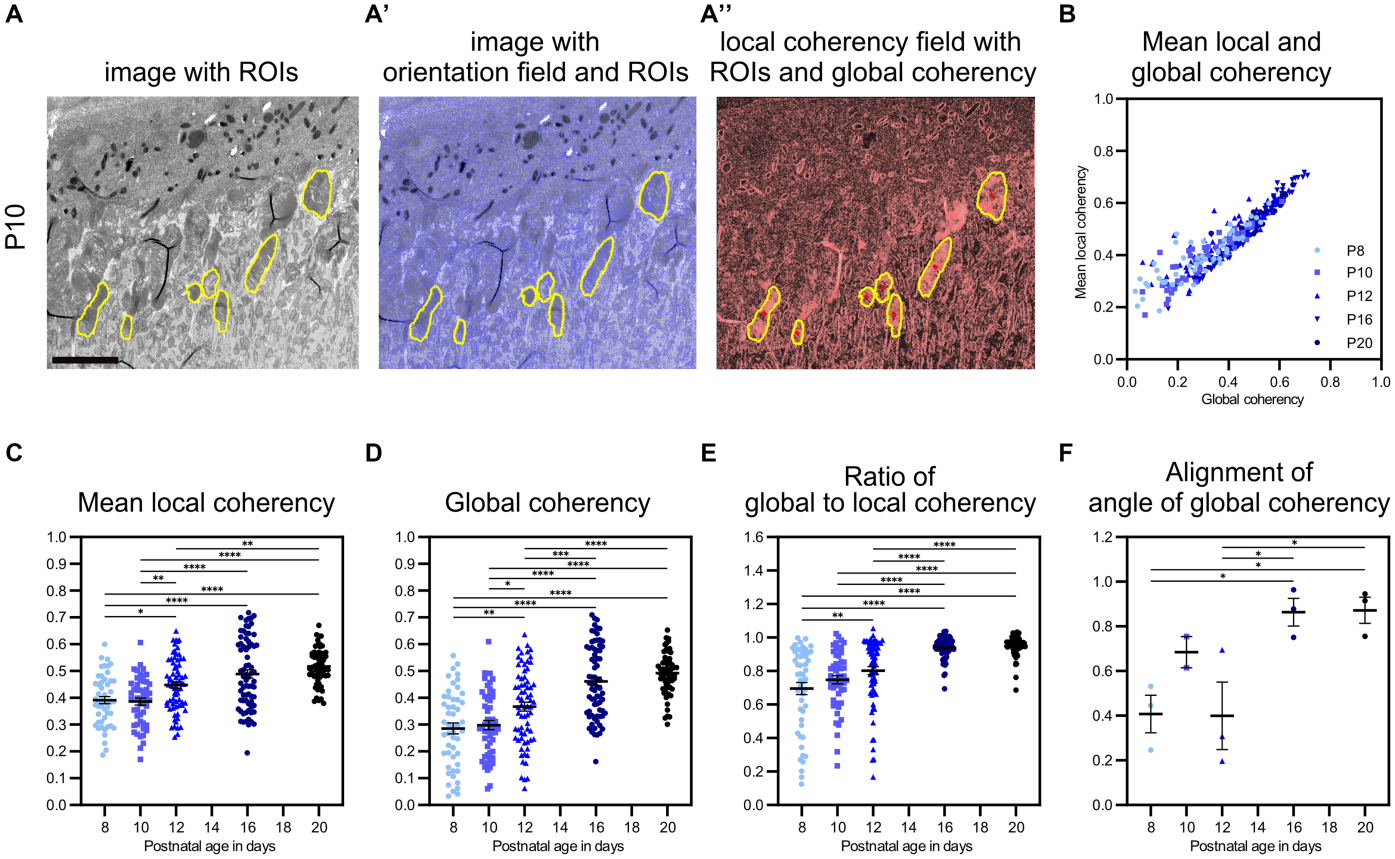
QuaPOS-TEM showed increasing coherency of POS disc membranes during the postnatal development of wild-type mice. **(A,A′,A″)** On selected grayscale TEM images of C57BL/6JRj (WT) retinas from postnatal age P8 to P20 (exemplary images shown for P10), ROIs were selected manually before the orientation and coherency fields were calculated. Scale bar = 5 μm. **(B–E)** Whole images were analyzed automatically, and mean local coherency, global coherency, and the angle of global coherency were retrieved for all selected ROIs. The ratio of global and local coherency was calculated per ROI. Plots show the values of individual ROIs (mean ± SEM, *n* = 2–3 with 12–30 ROIs each). An increase in local and global coherency of POS membranes over time was observed. **(F)** The alignment of the angle of global coherency was computed per biological replicate (mean ± SEM *n* = 2–3). **(C–F)** One-way analyses of the variance revealed significant dependency on postnatal age for all analyzed measures (*n* = 2–3, one-way ANOVA followed by a *post-hoc* Tukey test, * *p* < 0.05, ** *p* < 0.01, *** *p* < 0.001, **** < 0.0001).

In addition to the number and size of POS, their ultrastructural morphology is important for functionality. Healthy POS contain a massive amount of light-sensitive pigments that reside in hundreds of stacked membranous discs in rods or aligned plasma membrane evaginations in cones (Roepman and Wolfrum, 2007; Gilliam et al., 2012; Molday and Moritz, 2015; Goldberg et al., 2016; Chen et al., 2021). To quantify POS ultrastructure through membrane stack alignment on TEM images, the custom MATLAB code QuaPOS-TEM was developed. POS were selected manually as ROIs on TEM images of a developmental time series (P8–P20) of WT mouse retinal cross sections (Figures 3, 5; Supplementary Figure S5). Subsequently, whole images, including all annotated ROIs, were processed automatically.

First, orientation analysis was performed based on the image intensity gradient. Here, the convolutional Scharr operator (Scharr, 2000) optimized for minimal orientation bias was applied within a sliding window of 5 × 5 pixels centered around the query point (Figures 3A,A′, blue box and blue sticks). Membrane leaflets are heavily stained with osmium and therefore appeared dark on TEM images. For this reason, the local orientation of individual membrane layers was perpendicular to the image intensity gradient. Orientations were retrieved as nematic vectors and were shown as short blue sticks on each query point of the intensity image (Figures 3A–D). The resulting orientation field tracked the underlying membranous structures of POS with various membrane morphologies (Figures 3A–D).

Next, a coherency analysis was performed to evaluate whether the POS were stacked properly. The coherency measured the degree of alignment between orientations (nematic vectors) in a certain area of pixels and was computed from the *scalar nematic order parameter* (Soans et al., 2022, Figure 3A″, red box and red stick). A coherency value could range between 0 and 1 and was represented as a red stick with a certain length and direction (Figure 3A″). Whole images were analyzed, and two coherency values were retrieved per ROI (POS). On the one hand, the local coherency was analyzed in the vicinity of query points by computing the coherency in a box of 25 × 25 pixels (Figures 3A″–D″). The box size was chosen in order to span two to three membrane layers having a thickness of 0.02–0.03 μm each (Carter-Dawson and Lavail, 1979; Molday and Moritz, 2015; manually tested in a selection of images, data not shown). The mean of all local coherencies was calculated per ROI as mean local coherency (Figures 3E–G). On the other hand, the alignment of membrane stacks across complete ROIs was measured as global coherency by taking all pixels into account for which the 5 × 5 kernel of orientation analysis was fully contained within the given ROI (Figures 3B″–D″,E–G). The angle of global coherency was retrieved and provided the dominant direction of the membranes in the given POS (Figures 3E′–G′). Images and plots were created within the code to visualize ROIs (yellow contour), orientations (blue sticks), local coherencies (thin red sticks), and global coherencies (thick red sticks) (Figures 3B–D). Additionally, local coherencies and global coherency within each ROI were plotted as density functions and polar histograms respecting the 180-degree symmetry (Figures 3E–G).

The automated method QuaPOS-TEM was successfully applied to analyze the ultrastructure of POS on TEM images of a developmental time series (P8–P20) of WT mouse retinas (Figures 5B,D; Supplementary Figure S5). Different morphological types of POS membranes were identified. “Chaotic” POS, as present, for example, at P8, resulted in low global coherency (here 0.033, Figures 3B,B′,B″,E,E′). The local coherencies of the ROI accumulated in the left half of the density functions and pointed into various directions in the polar histogram (Figures 3E,E′). However, the mean local coherency yielded a value of 0.256, which indicated partly alignment of the membranes inside the ROI (Figure 3E). At P10, “partly stacked” POS could be observed and showed medium values for both global and local coherency (Figures 3C″,F). The mean local coherency was higher than the global coherency, clarifying that several regions of membranes are each well aligned within itself but less aligned to each other (Figures 3C,C′,C″,F,F′). The local coherencies are distributed over the whole density function plot, while the directions are accumulated in two quarters in the polar histogram (Figure 3F′). Stacked OS, here from P16, yielded high values for both mean local and global coherency (Figures 3D,D′,D″,G,G′), elucidating alignment of the membranes in the vicinity and across the whole ROI. The values of the density function shifted to the right, and the directions of the local coherencies overlapped quite well in the polar histogram (Figures 3G,G′). The angle of global coherency provided information about the dominant direction of the membranes within the POS (Figures 3E″,F″,G″). However, the angles of coherency depend on the position of the sample during the image acquisition. Therefore, they can only be compared between the POS of one sample and the imaging run.

During the development of QuaPOS-TEM, different parameters were considered. The method is dependent on the image resolution, which is determined by the magnification used during imaging and subsequent down-sampling at the level of the input grayscale image. The imaging magnification of 5,000× (287.5 pixels = 1 μm) was the compromise of providing high resolution while maintaining reasonable expenditure of imaging time. Down-sampling at the level of grayscale image or at the level of the orientation field was not used as it decreased accuracy of the membrane tracking (data not shown). However, a down-sampling by factor 2 could be used to process images obtained at a magnification of 10,000× providing a higher resolution (575 pixels = 1 μm). Furthermore, different box radii (6 and 24 pixels) for the local alignment were tested. The values for local coherency were slightly changed, but the tendency remained similar for POS of different morphologies (data not shown).

In summary, the code QuaPOS-TEM calculated three values for each ROI (POS) of a TEM image. The code enabled quantification of the alignment and morphology of membrane stacks both locally and globally within POS. POS with unaligned membrane stacks achieved low values for both coherency measurements (Figure 3E). POS with partly stacked membranes achieved a higher measurement for mean local coherency, while global coherency stayed medium (Figure 3F). POS with well-aligned membrane stacks got a higher value for both mean local and global coherency (Figure 3G). The angle of global coherency provided information about the dominant direction of the membranes within the POS (Figures 3E′,F′,G′).

#### 3.3.2 QuaPOS-TEM showed increasing coherency of POS membranes during postnatal development

After validating the performance of the method QuaPOS-TEM on individual POS of various morphology, the code was used to quantify the membrane alignment of POS during postnatal development (P8–P20) of WT animals on a set of whole images (Figures 5A,A′,A″; Supplementary Figure S5). 2–3 biological replicates with 12–30 ROIs (technical replicates) each were analyzed per time point. Mean local coherency, global coherency, and angle of global coherency were analyzed per ROI, mean and SEM were calculated for each developmental age (Figures 5B–E). One-way analyses of variance revealed significant dependency on postnatal age for both mean local coherency and global coherency (*F*(4, 299), *p*-value <0.0001, one-way ANOVA, Figures 5C,D). The mean local coherency increased from 0.39 at P8 to 0.52 at P20, while the global coherency raised from 0.29 at P8 to 0.49 at P20 (Figures 5C,D). Despite high variation within individual groups, *post-hoc* analysis by Tukey’s multiple comparison test showed significant differences between all groups except for P8 vs. P10, P12 vs. P16, and P16 vs. P20 for mean local coherency (Figure 5C). The same test resulted in significant differences between all groups except for P8 vs. P10 and P16 vs. P20 for global coherency (Figure 5D). Quantification by QuaPOS-TEM showed that POS membranes are already partly stacked during early postnatal development and increase to align in vicinity during maturation (Figure 5C). However, the overall alignment of membranes that was measured by global coherency changed stronger during postnatal development with an increase, especially between P10 and P16 (Figure 5D). While mean local coherency and global coherency appear different at P8 (a ratio of 0.69 between global and mean local coherency), they become more similar during maturation, with a ratio of 0.95 at P20 (Figure 5E). This convergence of both coherency values was significantly dependent on age (*F*(4, 299), *p*-value <0.0001, one-way ANOVA, Figures 5B,E). Therefore, the ratio of global coherency to mean local coherency evolved to be an indicator for POS maturation with significant differences between younger and older groups (*post-hoc* Tukey test, Figure 5E). The described comparisons were also analyzed after averaging all values per biological replicate and showed similar trends (Supplementary Figure S5).

To continue the analysis of coherency, the orientation of ROIs toward each other within the same biological replicate (images from one imaging run without movement of the specimen) was analyzed by comparing their angles of global coherency (Figure 5F). A significant change over time was revealed (*F*(4, 9), *p* = 0.0107, one-way ANOVA, Figure 5F). While the orientation of the angle of global coherency varied at P8 (0.41) and P12 (0.40), the POS membranes were oriented in similar directions at P16 (0.86) and P20 (0.87) across the images of individual samples (Figure 5F).

To sum up, quantification of POS with QuaPOS-TEM revealed an increased alignment of membranes in the vicinity, throughout individual POS, and across whole samples during postnatal development of WT animals. The mean local coherency, global coherency, ratio between global and mean local coherency, and alignment of the angle of global coherency efficiently characterized the maturation of POS.

#### 3.3.3 Quapos-TEM revealed defects in the coherency of POS membranes in the degeneration mouse models RhoKO and rd19

**Figure 6:**
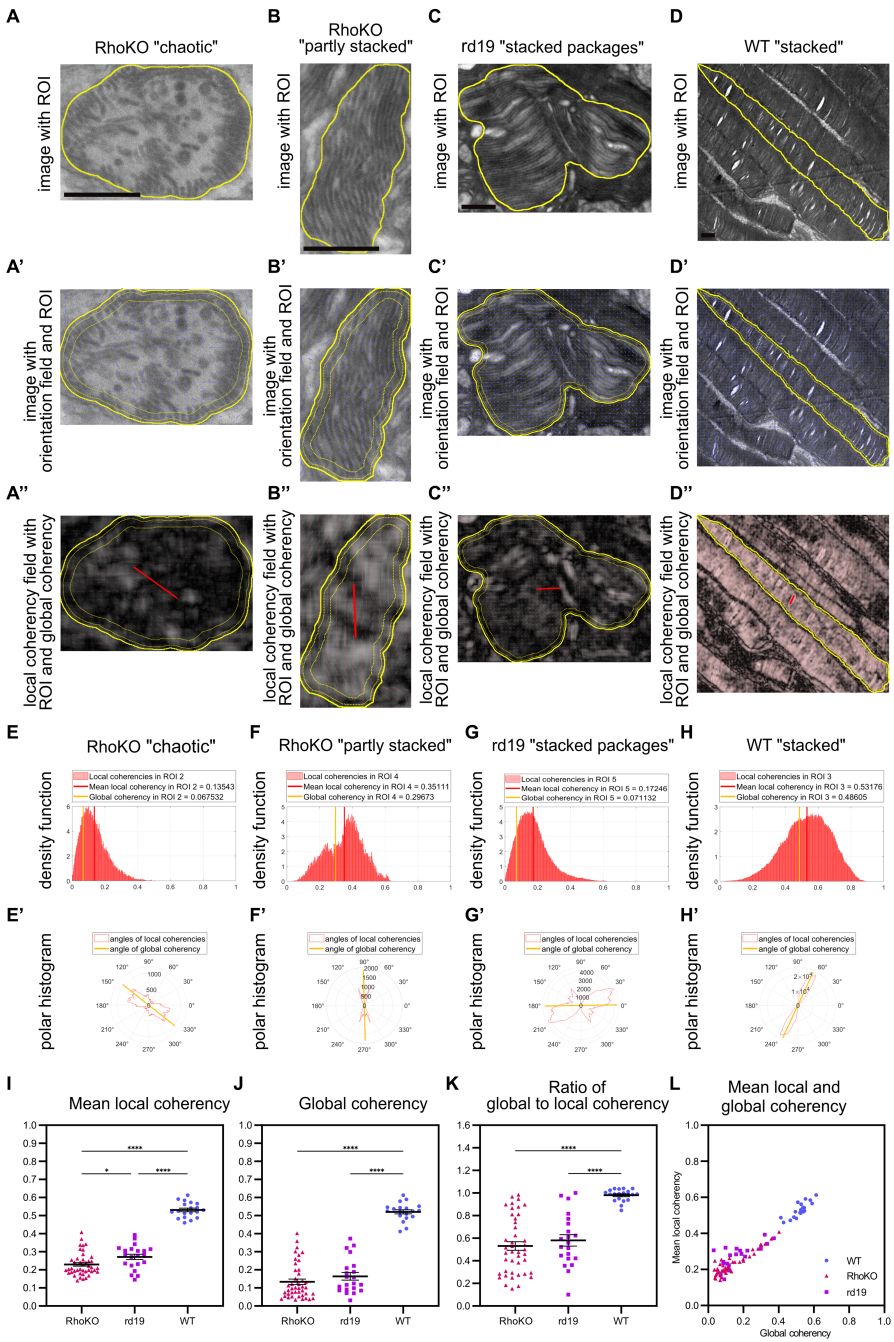
QuaPOS-TEM reveals defects in the coherency of POS membranes in the retinal degeneration mouse models RhoKO and rd19. **(A–D)** POS morphology is disturbed in RhoKO and rd19 retinas compared to WT mice on TEM images at 1 month of age. Scale bar = 0.5 μm. POS of RhoKO mice showed “chaotic” or “partly stacked” membrane morphology **(A,B)**. POS of rd19 mice contained “stacked packages” of membranes **(C)**. Control WT mice displayed POS with “stacked” membranes **(D)**. **(A′–D′,A″–D″)** Within selected ROIs (yellow contour), the orientation of the membranes was tracked as orientation field (blue sticks), and their coherency was computed. Local coherencies were visualized as thin red sticks of a certain length and angle and emphasized by background color **(A″–D″)**. Global coherencies were shown as thick red sticks **(A″–D″)**. **(E–H)** Density functions revealed the distribution of local coherencies (red areas) and the resulting mean local coherency (thick red line) and global coherency (thick orange line). The POS with “chaotic” membranes **(E)** or “stacked packages” of membranes **(G)** showed low coherency, the one with “partly stacked” membranes **(F)** resulted in medium, and the POS with “stacked” membranes yielded **(G)** high coherency values. **(E′–H′)** The directions of local coherencies within each ROI were plotted as polar histograms (red outlines) respecting the 180-degree symmetry and showed the computed angle of the global coherency (thick orange line). The polar histogram of the “chaotic” **(E′)** and “stacked packages” **(G′)** POS showed a variety of different coherency directions, while the “partly stacked” POS **(F′)** and “stacked” POS **(H′)** corresponded to (more) narrow distributions of coherency angles. **(I–L)** Several ROIs of RhoKO, rd19, and WT were analyzed automatically on selected TEM images. Mean local coherency, global coherency, and the angle of global coherency were retrieved, and the ratio of global to local coherency was calculated per ROI. **(J–L)** Plots show values of individual ROIs (mean ± SEM, *n* = 1–2 with 19–22 ROIs each). One-way analyses of variance revealed significant dependency on mouse line for all analyzed measures (one-way ANOVA followed by a *post-hoc* Tukey test, * *p* < 0.05, ** *p* < 0.01, *** *p* < 0.001, **** < 0.0001).

With this automated method in hand, the effects of retinal degeneration on POS membrane morphology were analyzed in two inherited retinal degeneration mouse lines (RhoKO, Humphries, 1997; and rd19, Chang et al., 2012) at 30 days of age. Both degeneration models show aberrant POS morphology on TEM images of retinal sections (Figures 6A–C; Supplementary Figure S6). POS of RhoKO animals displayed heterogeneous morphologies, with a mixture of small POS containing loose formations of mainly membrane vesicles (“chaotic”) or partly stacked membranes (Figures 6A,B; Supplementary Figure S6). POS of rd19 animals showed POS with curved, stacked packages of membranes of varying orientation (Figure 6C; Supplementary Figure S6).

Using the quantification method QuaPOS-TEM, the membrane alignment was quantified by analyzing mean local coherency, global coherency, and angle of global coherency (Figure 6). “Chaotic” POS present in the RhoKO mouse model resulted in a low global coherency (here 0.07) that elucidated a low overall alignment of membranes inside the ROI (Figures 6A,A′,A″,E). Similar to the “chaotic” POS from P8 in the WT animals, the local coherencies of the ROI accumulated in the left half of the density functions and pointed into various directions in the polar histogram (Figures 6E,E′). The mean local coherency yielded a value of 0.14, which indicated a low alignment of the membranes in the vicinity (Figures 6A,A′,A″,E). Similar values were achieved for POS-containing membrane vesicles (Supplementary Figure S6). The “partly stacked” POS that were observed in RhoKO animals showed medium values for both mean local coherency (0.35) and global coherency (0.30, Figures 6B,B′,B″,F). In retinal sections from rd19 animals, POS with “stacked packages” was observed, which yielded a low value for global coherency (0.07) and a higher but still low mean local coherency (0.18, Figures 6C,C′,C″,G). The density function of the local coherencies and the polar histogram resembled the ones from chaotic POS, indicating only partial alignment of membranes in the vicinity and overall low alignment of the membranes in the ROI. Other ROIs of the rd19 mouse line resulted in medium values for both mean local and global coherency as present for “partly stacked” POS during WT development (Supplementary Figure S6), elucidating partly alignment of the membranes in the vicinity and across the whole ROI. “Stacked” POS from control WT animals resulted in coherency values, density plots, and polar histograms similar to the ones from P16 and P20 in the developmental analysis (Figures 6D,D′,D″,H,H′).

In total, 17–43 ROIs (technical replicates) were analyzed on TEM images from 1 to 2 biological replicates per mouse line, and means plus SEM were calculated. Compared to WT controls, both degeneration mouse lines showed significantly decreased values for both mean local and global coherency [Figures 6I,J, *F*(2, 80), *p* < 0.0001, one-way ANOVA with *post-hoc* Tukey test]. The global coherency was affected more strongly, with a decrease from 0.52 for WT control animals to 0.13 for RhoKO and 0.16 for rd19 animals (Figures 6I,J). The mean local coherency shifted from 0.53 for the WT to 0.23 for the RhoKO and 0.27 for the rd19 mouse line (Figure 6I). This indicated that the POS membranes remained partly aligned in the vicinity during the degeneration of both mouse models. Interestingly, the POS of rd19 animals were significantly less affected than that of RhoKO animals, showing a slightly higher mean local coherency at 30 days of age (Figure 6I, *p* = 0.0278, *post-hoc* Tukey multiple comparison test). The ratio between global coherency and mean local coherency decreased significantly for RhoKO (0.53) and rd19 (0.58) compared to WT (0.98, Figures 6K,L). These values were even lower than the ratio observed at P8 during WT development, caused by the low values for global coherency observed (Figures 6J–L). All analyses were also conducted after averaging all values per biological replicate and showing similar trends (Supplementary Figure S6).

To gain further information about the alignment of membranes between individual ROIs, the angle of global coherency was analyzed and provided information about the dominant direction of the membranes within the separate ROIs (Figures 6E′,F′,G′,H′; Supplementary Figure S6). The dominant membrane direction was compared between all ROIs within one biological replicate by performing a coherency analysis between their angles of global coherency (Supplementary Figure S6). The alignment of POS of RhoKO animals was decreased to 0.52 for RhoKO animals and even further for the rd19 animal to 0.28 compared to the WT control (0.94, Supplementary Figure S6). However, statistical analyses could not be performed due to the small number of biological replicates.

In summary, QuaPOS-TEM was able to analyze POS of the two retinal degeneration mouse lines, RhoKO and rd19. The POS morphology was quantified and showed significant decreases in membrane alignment locally and globally inside the individual POS for both models.

## 4 Discussion

While important insights about POS formation and morphology were generated through manual analyses, such approaches are time-consuming, and therefore sample size is limited. Here, two automated bio-image analysis methods to quantify POS formation are presented. First, a supervised machine learning model was established and validated for automated segmentation of cone POS on cryosections stained for S-opsin and imaged by LM. Subsequently, the number of POS and different size, shape, and intensity parameters were quantified from three-dimensional image stacks (QuaPOS-LM). Second, a method to quantify the morphology of POS membranes based on the image gradient of TEM pictures was established. This method allowed to quantify the quality of POS at the ultrastructural level (QuaPOS-TEM). Evidence is provided that both methods can be used to analyze the developmental and degenerative processes of POS.

The chosen development timeframe (P4–P24) spanned prior to the onset of POS formation until their maturation and covered the eye opening between P12 and P14 (Zieske, 2004; Koehler et al., 2011). The samples represented different developmental stages and were suitable to establish quantification methods for POS with diverse morphologies. Here, POS were detected from P8 onwards during the postnatal development of C57BL/6JRj mice. Previously, it was shown that POS of cones rapidly increased between P5 and P6 and continued to rise until P11 in the central retina (Daum et al., 2017). The discrepancy between the onset of POS formation might be due to different mouse strains used (Gong et al., 2003; Siegert et al., 2009; Daum et al., 2017).

At various postnatal development stages, single cone POS were identified by QuaPOS-LM. However, later developmental stages frequently included segmentation results of clusters (predictions that correspond to several POS) or results of objects that were too small to be considered POS. Here, these objects were removed by setting a threshold based on the 5th and 95th percentiles of the measured volume. A watershed algorithm was tried to separate clusters of segmentation into single POS. However, the approach was judged unsuccessful since elongated objects were separated in both directions, vertically and horizontally (data not shown). Furthermore, small POS with a volume above the 5th percentile were observed in adult postnatal development timepoints. These POS showed a similar morphology as seen in early development stages and were included in the analysis. Unexpectedly, this led to smaller average values of the POS length in comparison to published results (Carter-Dawson and Lavail, 1979; Fu and Yau, 2007). These signals might result from phagocytosed POS fragments from the RPE or partial sectioning and other artifacts from tissue preparation. Such limitations might be circumvented by more sophisticated protocols such as optical tissue clearing (Henning et al., 2019; Cai et al., 2023) or improved segmentation models. Machine learning models could be based on a deep learning architecture such as stardist (Weigert et al., 2020) or cellpose (Stringer et al., 2021), which are designed to separate objects in close proximity. Generally, such models perform better than classic machine learning models and show increased values regarding true positive and true negative pixel readings. However, calculating the performance of the classifier established here with an initially defined test dataset resulted in a Jaccard index of over 70% within human agreement. Importantly, unlike manual assessment, the prediction of the classifier is reproducible and scalable for analyzing large amount of image data.

Given the correct representation of POS predicted by QuaPOS-LM, the method was applied to analyze POS formation throughout postnatal development in WT (P8–P24) and degeneration in Cpfl1 mice (between 8 days and 35 weeks of age). Throughout postnatal development, a positive correlation between age and POS number, summed volume, and length was observed, confirming the robustness of QuaPOS-LM with recent manually quantified results (Sharma et al., 2003; Daum et al., 2017). Furthermore, the method could be applied to analyze various size, shape, and intensity parameters of POS as well. Hence, the data revealed an overall increase in POS size throughout postnatal development. An opposite effect was observed when analyzing POS from Cpfl1 mice in comparison to age-matched WT control animals. Whereas some cone POS remained in adult Cpfl1 mice, a decline in number was observed until 35 weeks of age. Additionally, the Pearson’s R correlation coefficient indicated that the remaining cone POS of Cpfl1 mice are similar in regard to their size and morphology in comparison to WT control animals and hence properly formed, confirming previous results (Chang et al., 2002, 2009; Santos-Ferreira et al., 2015). Whether these cones are further degenerating after 35 weeks of age might be assessed in future studies.

Unlike cones, rod photoreceptors are much more abundant in the mouse retina (Carter-Dawson and Lavail, 1979). Hence, single-cell resolution for rods is challenging in LM, and thus QuaPOS-LM analysis on cryosections is not possible. However, the provided analysis method could be used to further characterize retinal development and degeneration of cone photoreceptors. Plus, the model could also be adapted to similar markers such as mid-wavelength (M) opsin to further analyze different photoreceptor types in development and disease or the distribution of S- and M-cones along the dorsoventral axis (Nadal-Nicolás et al., 2020).

While the number and volume of POS are important indicators for photoreceptor maturation and hence function, the proper alignment of membranous discs is also essential (Mustafi et al., 2009; Burgoyne et al., 2015; Molday and Moritz, 2015; Lewandowski et al., 2022). Membrane stacks become visible at high magnification by TEM. In this study, TEM images of WT mice mainly containing rod POS were used to establish an ultrastructural quantification measurement for alignment of POS membranes. Single POS were analyzed using the custom-written MATLAB code QuaPOS-TEM to extract the orientation of membranes, their coherency (alignment), and dominant direction. Manual selection of POS as separate ROIs was performed before automated analysis by QuaPOS-TEM using ImageJ. Ideally, the automated selection of ROIs should be used for the generation of unbiased datasets. However, currently, there are no appropriate techniques established to perform instance segmentation on EM images, especially in the case of highly crowded structures (Aswath et al., 2023). Deep learning approaches such as convolutional neural networks have emerged as the preferred option for automated feature extraction and segmentation in EM data, as reviewed by Aswath et al. (2023). However, they state that “instance segmentation of apposing organelles, especially when the organelles contain structures that are similar to their membranes,” is still challenging (Aswath et al., 2023). POS consist of layers of electron-dense and electron-thin regions created by the typical stacking of membranes. This hampers POS segmentation in an automated way and should be addressed in future studies.

In this context, it might be helpful to combine the methods of QuaPOS-LM and QuaPOS-TEM to select regions of interest by correlative light and electron microscopy (CLEM). However, for the two techniques shown here, different sample preparation methods were used (cryostat sections vs. epoxy resin sections), which rules out a correlative workflow. Alternatively, an on-section immunolabeling approach using methacrylate or Tokuyasu cryosections could provide a correlative workflow (Eberle et al., 2012; Fabig et al., 2012; Santos-Ferreira et al., 2015; Völkner et al., 2021, 2022), but the membrane contrast would be either weak (methacrylate, lipids not fixed with osmium and therefore partially extracted) or inverted (Tokuyasu cryosectioning), rendering the adaptation of the analysis methods shown here difficult. Another option to combine QuaPOS-LM and -TEM in a correlative workflow would be to process the cryostat sections after LM imaging for TEM (Sousa et al., 2021). Although technically demanding, this method provides sufficient ultrastructure and similar contrast to the images presented here (Sousa et al., 2021, unpublished data).

During the development of the QuaPOS-TEM code, different parameters were tested to optimize analysis. The code QuaPOS-TEM was optimized for our application. It is important to note that it can be adapted for other applications to analyze, for example, images of different resolutions. However, a calculation of parameters and adjustment at the appropriate position in the MATLAB code would be required to be comparable to the presented analyses (e.g., for images with a resolution of 575 pixels = 1 μm, dsG or dsO with factor 2, or a local box radius of 24 pixels would need to be used).

During the preparation of ultrathin sections of the retinal tissue for TEM, the introduction of cutting artifacts could especially cause problems regarding proper POS morphology. Whereas partial sectioning could influence measurements about POS size, the established QuaPOS-TEM method is independent from that. Hence, it might be preferred to measure POS volume, length, diameter, and size on serial block-face EM images rather than conventional TEM. Here, regions were selected where the RPE was closely attached to the POS for imaging. POS with obvious interruptions were excluded during the manual selection of ROIs. Additionally, it needs to be considered that the membrane structure was influenced by the fixative used during tissue preparation (Ding et al., 2015). Given such typical variations due to tissue preparation and sectioning, it is of utmost importance to increase sample size and thus make use of automated analysis.

After orientation analysis of grayscale images, global coherency and mean local coherency measures resulted in values between 0 and 0.7. The local coherency was used to quantify the alignment of membrane stacks in the vicinity, which resulted to be higher than the global coherency for most ROIs, especially when an ROI contained several regions that are each well aligned within itself but less aligned to each other. The box size for local alignment was chosen in order to span two to three membrane layers having a thickness of 0.02–0.03 μm each (Carter-Dawson and Lavail, 1979; Molday and Moritz, 2015; manually testified in a selection of images, data not shown). Similar trends were found for box sizes of half or double the size (data not shown). However, different box sizes can be used in other applications by manually changing the MATLAB code. Notably, we did not observe the maximum value of 1 for neither global nor mean local coherency in any sample. This was expected as it would indicate perfect alignment within a POS and within the complete patch of 25 × 25 pixels in size.

Finally, QuaPOS-TEM was applied as a tool for quantifying defects in POS membrane formation and disc orientation in two inherited retinal degeneration mouse lines at 1 month of age. Indeed, significantly decreased local and global coherency for both the rd19 and RhoKO mouse models were detected. In the RhoKO model, the POS morphology was affected stronger by lower mean local coherency. This analysis example proved applicability of QuaPOS-TEM and paves the way for investigating degenerative processes of POS quantitatively on an ultrastructural level in various disease models and over time.

## 5 Conclusion

In conclusion, automated image segmentation on cryosections stained for S-opsin by a classifier based on machine learning (QuaPOS-LM) and analysis of the orientation of membrane stacks (QuaPOS-TEM) allow robust quantitative evaluation of POS formation and quality. Applying both methods, developmental and degenerative processes were successfully evaluated in the retina of WT mice and three retinal degeneration mouse models, respectively, using both LM and TEM images. The presented unbiased approaches are useful for in-depth analysis of POS in developmental studies, for disease modeling, or after therapeutic interventions affecting photoreceptors.

## Data availability statement

The datasets presented in this study can be found in online repositories. The names of the repository/repositories and accession number(s) can be found below: GitHub repository (https://doi.org/10.5281/ZENODO.10794251) and BioImage Archive repository (https://www.ebi.ac.uk/biostudies/bioimages/studies/S-BIAD1078).

## Ethics statement

The animal studies were approved by Ethics Committees of the Technische Universität Dresden and the Landesdirektion Sachsen, Germany. The studies were conducted in accordance with the local legislation and institutional requirements. Written informed consent was obtained from the owners for the participation of their animals in this study.

## Author contributions

SS: Conceptualization, Data curation, Formal analysis, Funding acquisition, Investigation, Methodology, Project administration, Software, Validation, Visualization, Writing – original draft. FS: Data curation, Formal analysis, Funding acquisition, Investigation, Methodology, Software, Validation, Visualization, Writing – original draft. KH: Data curation, Formal analysis, Methodology, Software, Validation, Visualization, Writing – original draft. TK: Data curation, Investigation, Resources, Writing – review & editing. IS: Methodology, Resources, Supervision, Writing – review & editing. RH: Formal analysis, Methodology, Software, Supervision, Writing – review & editing. MA: Conceptualization, Funding acquisition, Resources, Supervision, Validation, Writing – original draft.

## Footnotes

^1^ https://janvier-labs.com/en/fiche_produit/3_aged_c57bl-6jrj_mouse/

## Supplementary material

SUPPLEMENTARY FIGURE S1 Analysis of POS with LM and TEM. (A) Representative images of light microscopy images stained with the cone POS marker S-opsin (magenta), the pan-POS marker Peripherin (green), and DAPI (gray) from P4–P24. Peripherin^+^ labels were present from P6, while S-opsin^+^ POS were present from P8 onwards. Both markers indicated a change of POS number, size, and morphology throughout postnatal development (scale bar = 50 μm). (B, B′) Ultrastructural investigation of POS using TEM indicated changes in membrane stack organization throughout postnatal development. Unorganized POS membrane stacks become present around P8. POS became more frequently with rising age and indicated a change in their membrane stack organization over time.

SUPPLEMENTARY FIGURE S2 Different image operations were used by QuaPOS-LM. (A) Prior to the application of QuaPOS-LM the image dataset was normalized. The operations included a background subtraction using a top-hat-filter and normalization of the intensity based on the maximum pixel value found in the image (scale bar = 10 μm). (A′) The normalized images were filtered by the random forest classifier. The classifier can choose from three different filter operations including a Gaussian blur filter (Gaussian), a difference of Gaussian blur filter (DoG), or a Laplace box of Gaussian blur filter (LoG), all with a respective sigma of 1. (B) Statistics of the random forest classifier showed the usage of each filter by the model. (C) Problems of the random forest classifier: Sometimes POS, predominantly in older postnatal ages, are predicted as 1 label (magenta arrowheads; scale bar = 25 μm).

SUPPLEMENTARY FIGURE S3 Quantification of POS using QuaPOS-LM showed a change in morphology and number over time. (A) Heatmap representation of Pearson’s R correlation coefficients showed the relationships of all features with each other. (A′) Correlation vector of the age showed which features correlate with the postnatal age. Different size features revealed the highest Pearson’s R correlation coefficients. Among them were the summed POS volume and bounding box height. Average and boxplot representation of the (B, B′) POS Feret diameter, (C, C′) minimum intensity, and (D, D′) sphericity showed changes throughout postnatal development (*n* = 3–4, *N* = 1, One-way ANOVA followed by a post-hoc Tukey test, * < 0.05, ** < 0.01, *** < 0.001). Whiskers of the boxplots indicate the 1.5 interquartile range. (E, F, G, and H) Heatmap annotation of selected features at two different timepoints throughout postnatal development showed a visual representation of the POS change observed (scale bar = 25 μm).

SUPPLEMENTARY FIGURE S4 Cone POS are impaired in Cpfl1 mice. Direct comparison of age-matched (A) C57BL/6JRj (WT) and (B) Cpfl1 mice indicated a reduction in cone POS number as well as impaired morphology in adult Cpfl1 mice (scale bar = 50 μm).

SUPPLEMENTARY FIGURE S5 Coherency analysis of POS membranes during postnatal development of wild-type mice by QuaPOS-TEM. (A to A″, B to B″, C to C″, D to D″, E to E″) On selected grayscale TEM images of C57BL/6JRj (WT) retinas from P8 to P20 ROIs were selected manually before the orientation and coherency fields were calculated. Scale bar = 5 μm. (F, G, H) Whole images were analyzed automated and mean local coherency, global coherency and the ratio of global to local coherency were retrieved for all selected ROIs. Values were averaged per biological replicate (mean ± SEM, *n* = 2–3 with 12 to 30 ROIs each). One-way analyses of the variance revealed significant dependency on postnatal age for the ratio of global to local coherency (*n* = 2–3, One-way ANOVA followed by a post-hoc Tukey test, * *p* < 0.05, ** *p* < 0.01, *** *p* < 0.001, **** < 0.0001).

SUPPLEMENTARY FIGURE S6 Coherency analysis by QuaPOS-TEM of POS membranes in the degeneration mouse models RhoKO and rd19. (A, B, E, F, G) Disturbed POS morphology was observed in RhoKO and rd19 retinas when compared to WT mice using TEM images at 1 month of age. Scale bar = 0.5 μm (A, B) and 5 μm (E, F, G). POS of RhoKO mice contained membrane “vesicles” (A). POS of rd19 mice contained “partly stacked” of membranes (B). (A′, A″, B′, B″) Within selected ROIs (yellow contour) the orientation of the membranes was tracked as orientation field (blue sticks) and their coherency was computed. Local coherencies were visualized as thin red sticks of a certain length and angle and emphasized by background color (A″, B″). Global coherencies were shown as thick red sticks (A″, B″). (C, D) Density functions revealed the distribution of local coherencies (red areas) and the resulting mean local coherency (thick red line) and global coherency (thick orange line). (C′, D′) The directions of local coherencies within each ROI were plotted as polar histograms (red outlines) respecting the 180-degree-symmetry and showed the computed angle of the global coherency (thick orange line). (H, I, J, K) Several ROIs of RhoKO, rd19 and WT were analyzed automated on selected TEM images. Mean local coherency, global coherency and the angle of global coherency were retrieved and the ratio of global to local coherency was calculated per ROI. Values were averaged per biological replicate (mean ± SEM, *n* = 1–2 with 19 to 22 ROIs each).
