# Supplementary figures and images for: Automated quantification of photoreceptor outer segments in developing and degenerating retinas on microscopy images across scales
Source: Front Mol Neurosci. 2024 May 24;17:1398447. doi: 10.3389/fnmol.2024.1398447 (PMC11157083; doi:10.3389/fnmol.2024.1398447)

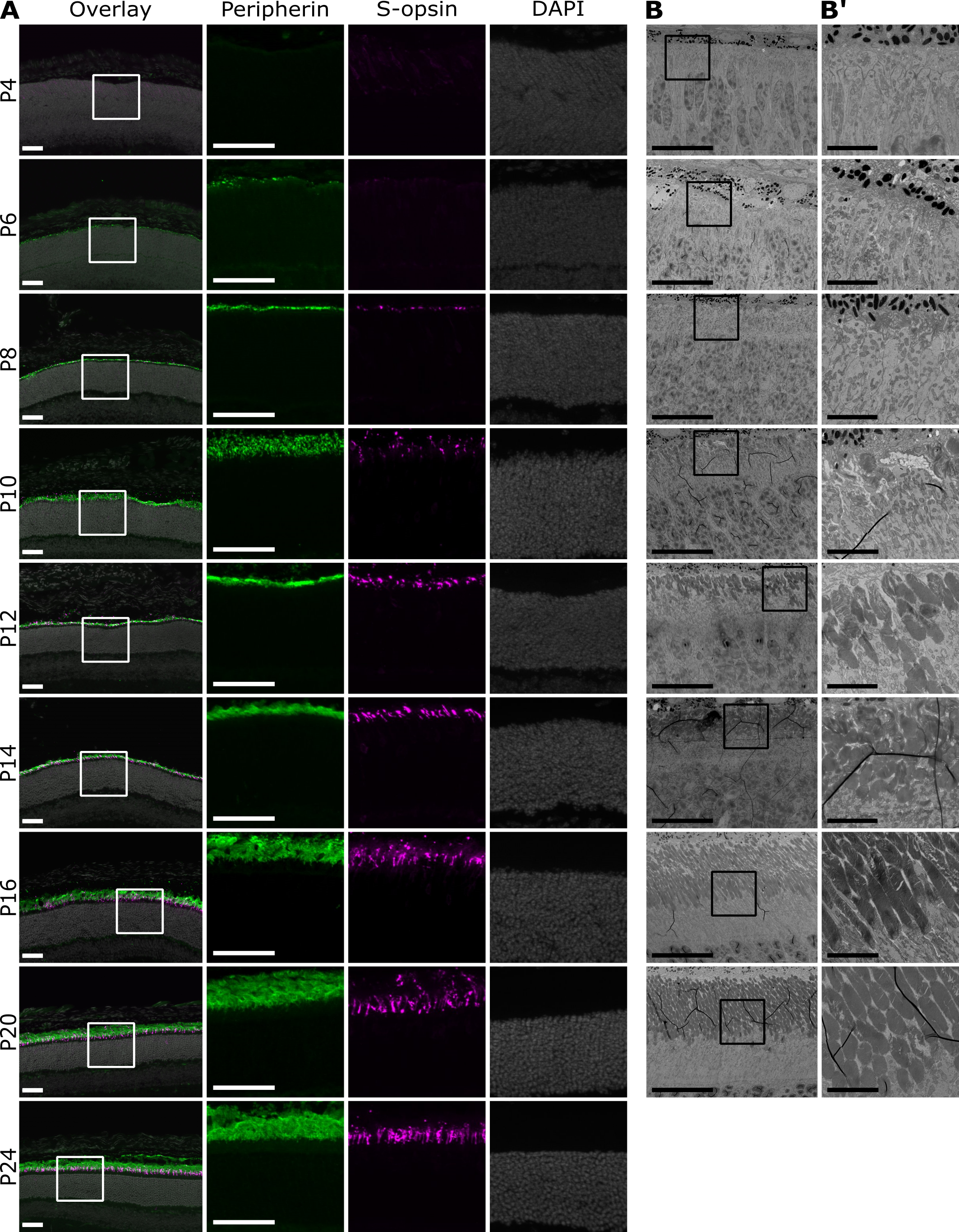

Supplement: SUPPLEMENTARY FIGURE S1 — Analysis of POS with LM and TEM. (A) Representative images of light microscopy images stained with the cone POS marker S-opsin (magenta), the pan-POS marker Peripherin (green), and DAPI (gray) from P4–P24. Peripherin+ labels were present from P6, while S-opsin+ POS were present from P8 onwards. Both markers indicated a change of POS number, size, and morphology throughout postnatal development (scale bar = 50 μm). (B, B′) Ultrastructural investigation of POS using TEM indicated changes in membrane stack organization throughout postnatal development. Unorganized POS membrane stacks become present around P8. POS became more frequently with rising age and indicated a change in their membrane stack organization over time. [file Image_1.TIFF]

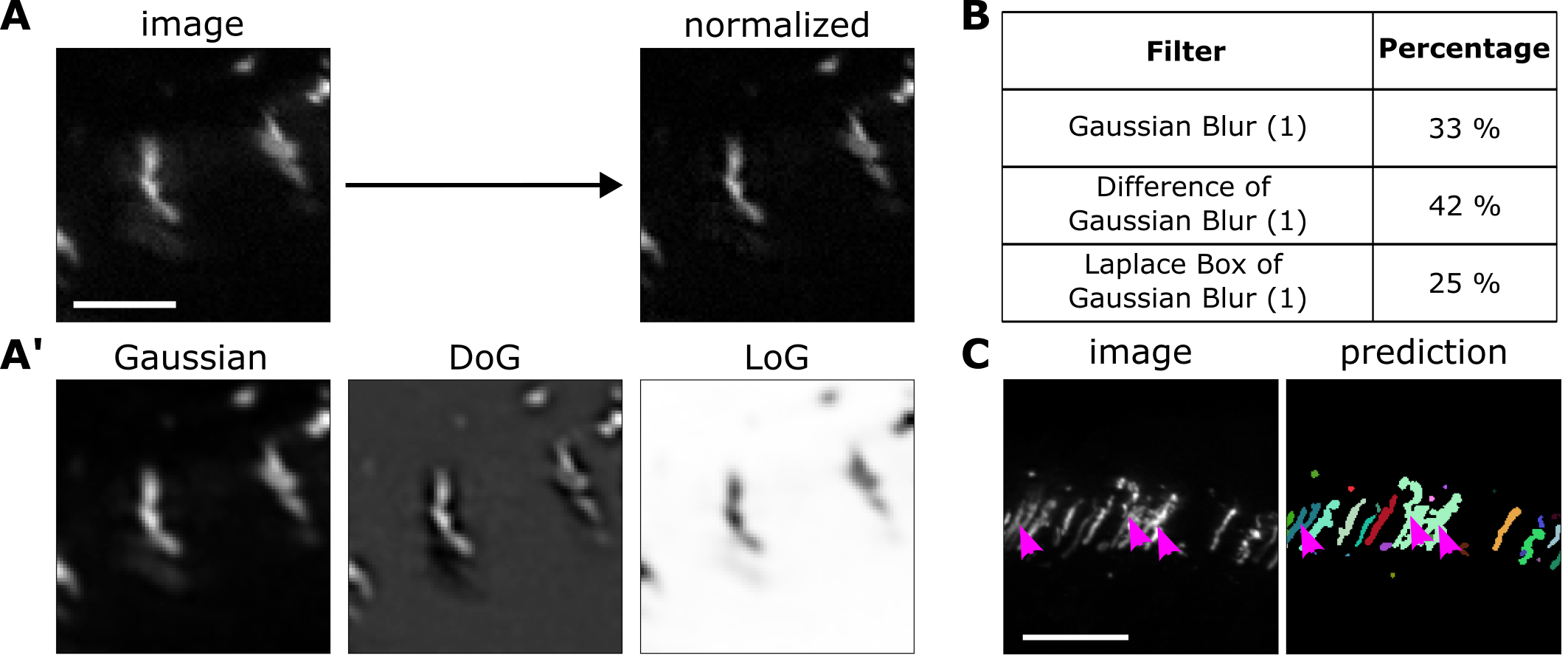

Supplement: SUPPLEMENTARY FIGURE S2 — Different image operations were used by QuaPOS-LM. (A) Prior to the application of QuaPOS-LM the image dataset was normalized. The operations included a background subtraction using a top-hat-filter and normalization of the intensity based on the maximum pixel value found in the image (scale bar = 10 μm). (A′) The normalized images were filtered by the random forest classifier. The classifier can choose from three different filter operations including a Gaussian blur filter (Gaussian), a difference of Gaussian blur filter (DoG), or a Laplace box of Gaussian blur filter (LoG), all with a respective sigma of 1. (B) Statistics of the random forest classifier showed the usage of each filter by the model. (C) Problems of the random forest classifier: Sometimes POS, predominantly in older postnatal ages, are predicted as 1 label (magenta arrowheads; scale bar = 25 μm). [file Image_2.tiff]

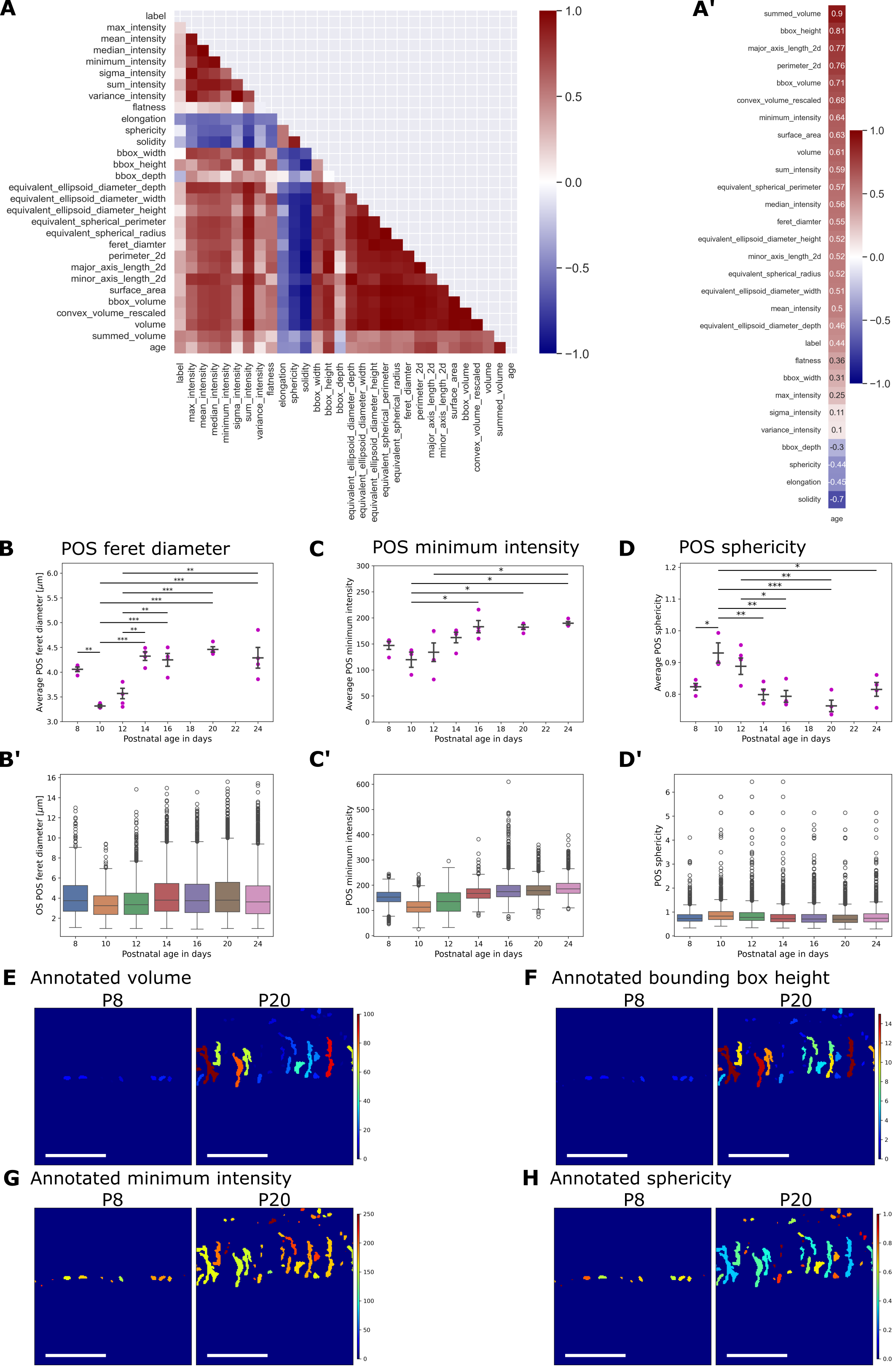

Supplement: SUPPLEMENTARY FIGURE S3 — Quantification of POS using QuaPOS-LM showed a change in morphology and number over time. (A) Heatmap representation of Pearson’s R correlation coefficients showed the relationships of all features with each other. (A′) Correlation vector of the age showed which features correlate with the postnatal age. Different size features revealed the highest Pearson’s R correlation coefficients. Among them were the summed POS volume and bounding box height. Average and boxplot representation of the (B, B′) POS Feret diameter, (C, C′) minimum intensity, and (D, D′) sphericity showed changes throughout postnatal development (n = 3–4, N = 1, One-way ANOVA followed by a post-hoc Tukey test, * < 0.05, ** < 0.01, *** < 0.001). Whiskers of the boxplots indicate the 1.5 interquartile range. (E, F, G, and H) Heatmap annotation of selected features at two different timepoints throughout postnatal development showed a visual representation of the POS change observed (scale bar = 25 μm). [file Image_3.TIFF]

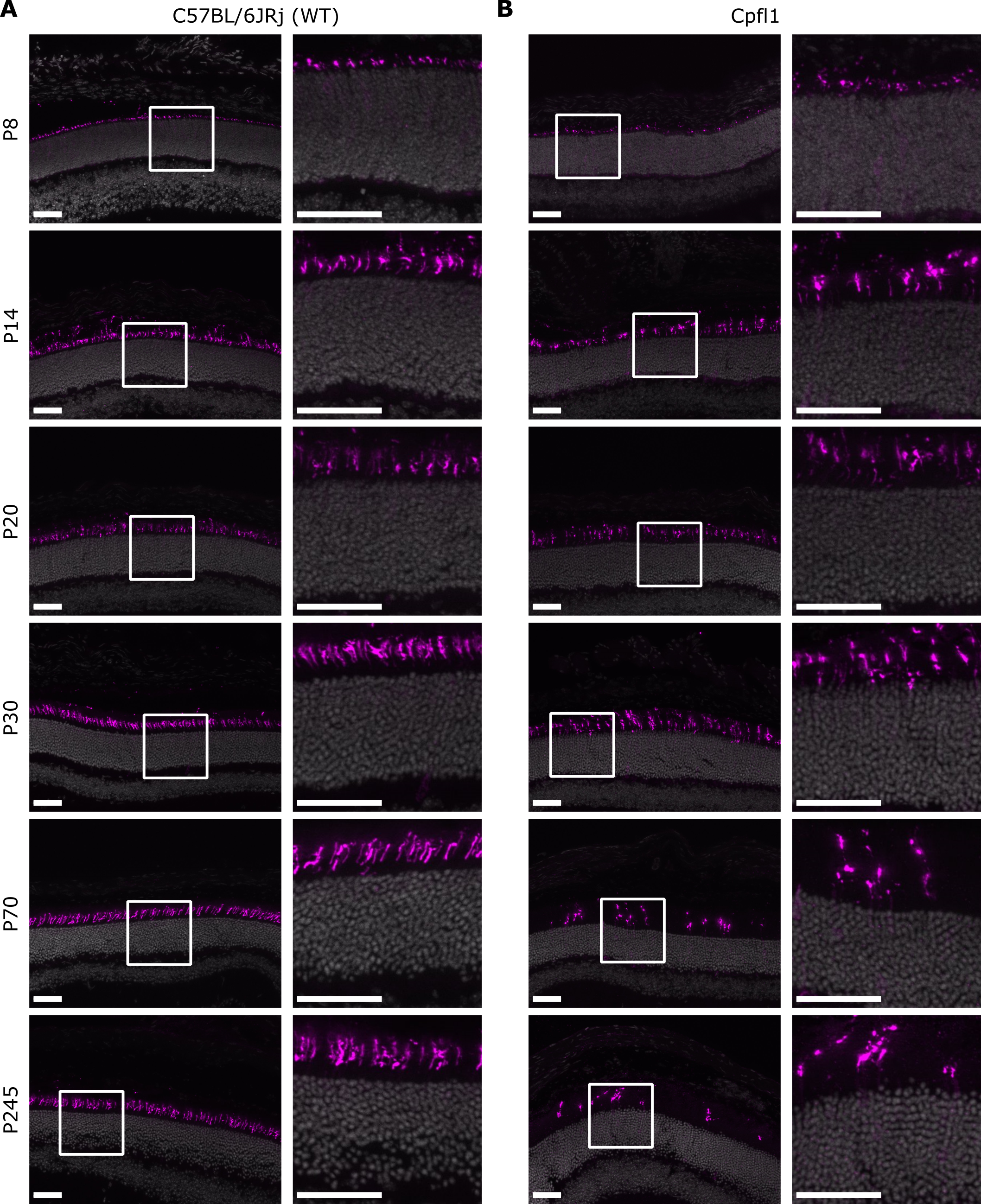

Supplement: SUPPLEMENTARY FIGURE S4 — Cone POS are impaired in Cpfl1 mice. Direct comparison of age-matched (A) C57BL/6JRj (WT) and (B) Cpfl1 mice indicated a reduction in cone POS number as well as impaired morphology in adult Cpfl1 mice (scale bar = 50 μm). [file Image_4.TIFF]

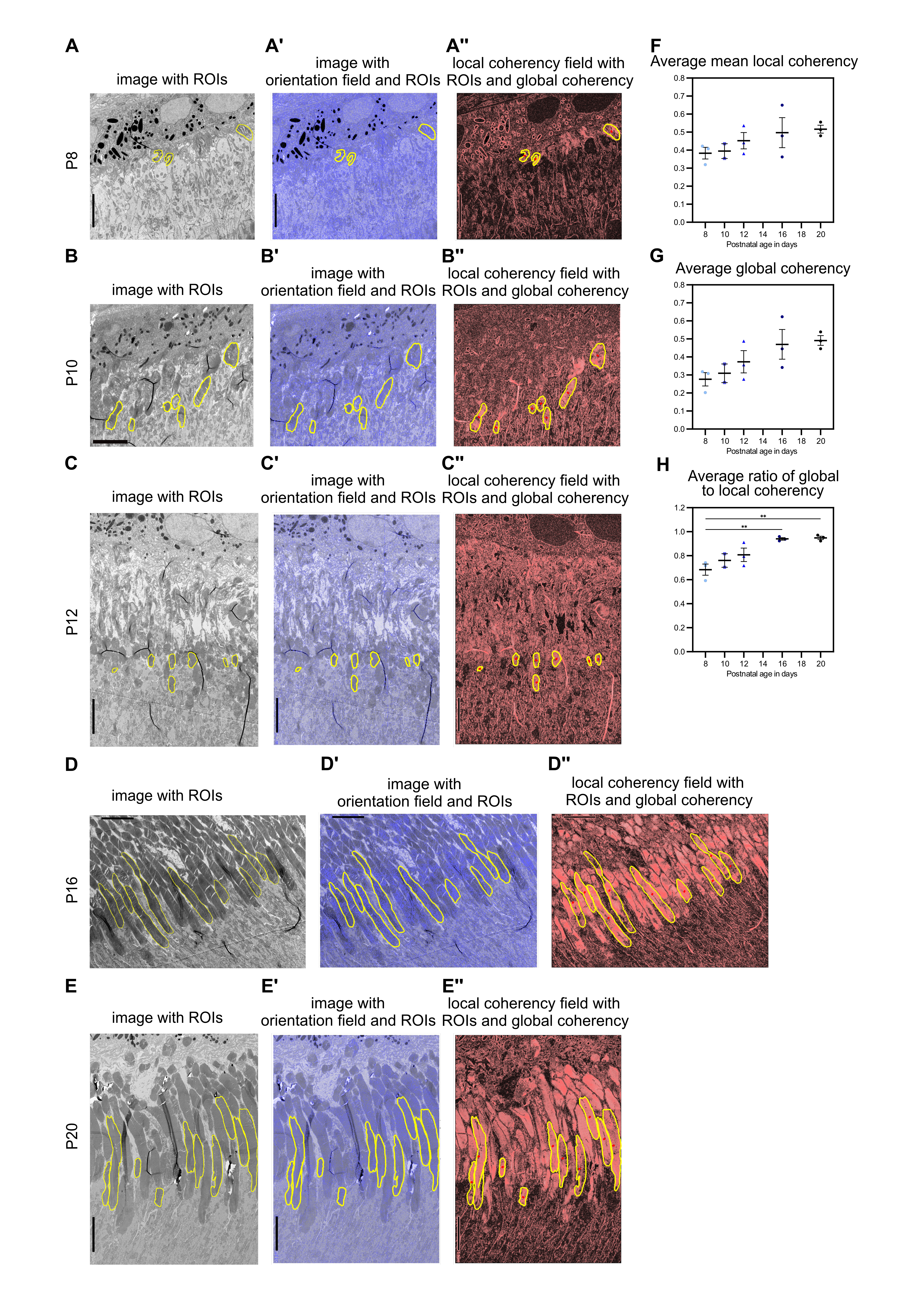

Supplement: SUPPLEMENTARY FIGURE S5 — Coherency analysis of POS membranes during postnatal development of wild-type mice by QuaPOS-TEM. (A to A″, B to B″, C to C″, D to D″, E to E″) On selected grayscale TEM images of C57BL/6JRj (WT) retinas from P8 to P20 ROIs were selected manually before the orientation and coherency fields were calculated. Scale bar = 5 μm. (F, G, H) Whole images were analyzed automated and mean local coherency, global coherency and the ratio of global to local coherency were retrieved for all selected ROIs. Values were averaged per biological replicate (mean ± SEM, n = 2–3 with 12 to 30 ROIs each). One-way analyses of the variance revealed significant dependency on postnatal age for the ratio of global to local coherency (n = 2–3, One-way ANOVA followed by a post-hoc Tukey test, * p < 0.05, ** p < 0.01, *** p < 0.001, **** < 0.0001). [file Image_5.TIFF]

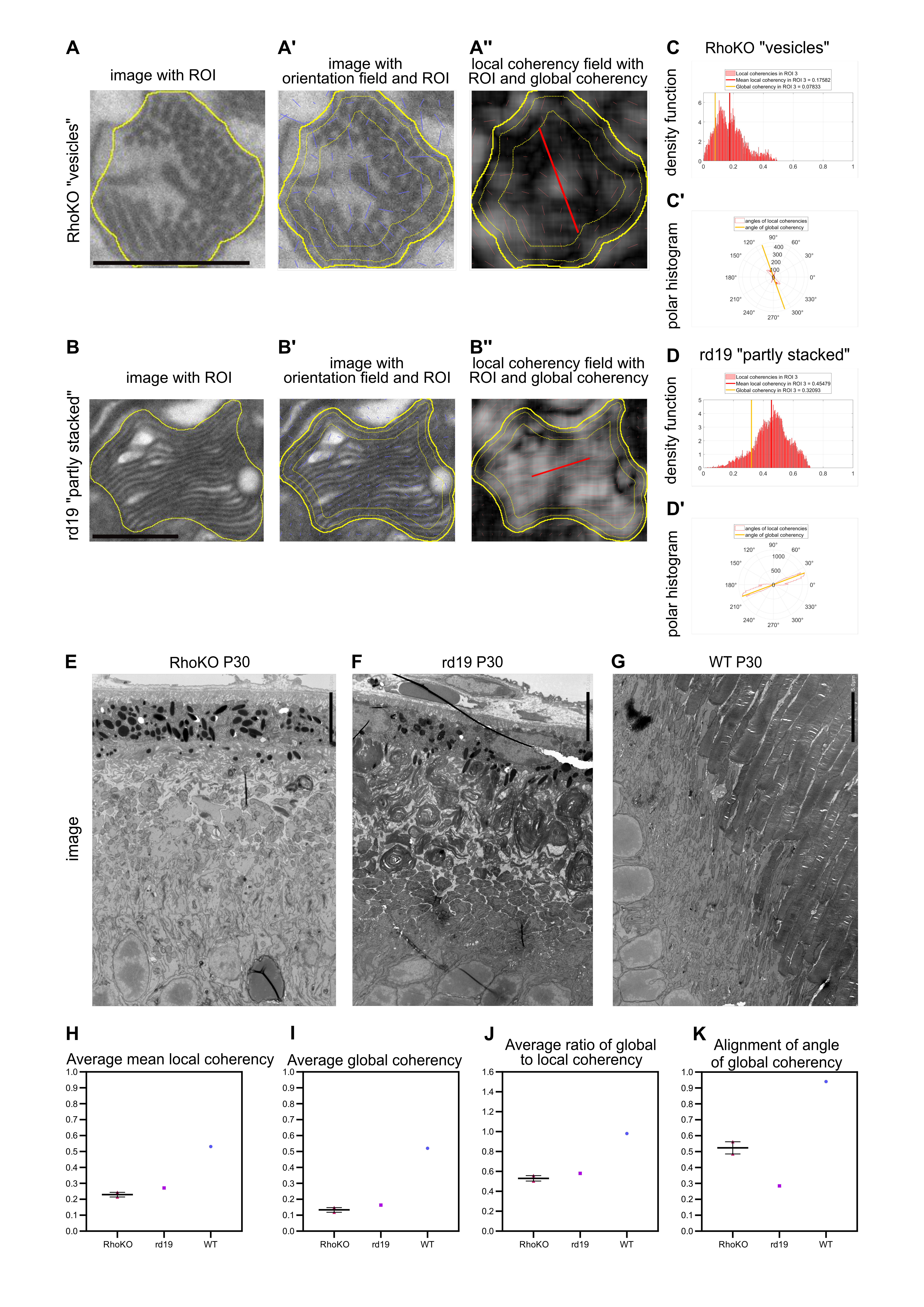

Supplement: SUPPLEMENTARY FIGURE S6 — Coherency analysis by QuaPOS-TEM of POS membranes in the degeneration mouse models RhoKO and rd19. (A, B, E, F, G) Disturbed POS morphology was observed in RhoKO and rd19 retinas when compared to WT mice using TEM images at 1 month of age. Scale bar = 0.5 μm (A, B) and 5 μm (E, F, G). POS of RhoKO mice contained membrane “vesicles” (A). POS of rd19 mice contained “partly stacked” of membranes (B). (A′, A″, B′, B″) Within selected ROIs (yellow contour) the orientation of the membranes was tracked as orientation field (blue sticks) and their coherency was computed. Local coherencies were visualized as thin red sticks of a certain length and angle and emphasized by background color (A″, B″). Global coherencies were shown as thick red sticks (A″, B″). (C, D) Density functions revealed the distribution of local coherencies (red areas) and the resulting mean local coherency (thick red line) and global coherency (thick orange line). (C′, D′) The directions of local coherencies within each ROI were plotted as polar histograms (red outlines) respecting the 180-degree-symmetry and showed the computed angle of the global coherency (thick orange line). (H, I, J, K) Several ROIs of RhoKO, rd19 and WT were analyzed automated on selected TEM images. Mean local coherency, global coherency and the angle of global coherency were retrieved and the ratio of global to local coherency was calculated per ROI. Values were averaged per biological replicate (mean ± SEM, n = 1–2 with 19 to 22 ROIs each). [file Image_6.TIFF]
